# Modulating the Physicochemical Properties of Mesoporous Strontium Silicate Nanostructures Using a Design-of-Experiments Approach

**DOI:** 10.3390/jfb17100490

**Published:** 2026-09-29

**Authors:** Dhiraj Kumar, Taruna Singh, Tristen Nies, Grace S. Liu, Maycoll Johnson, David Mei, Wandi Gu, Conrado Aparicio, Isha Mutreja, Robert S. Jones

**Affiliations:** 1 Division of Basic Sciences, Department of Diagnostic and Biological Sciences, University of Minnesota, Moos Health Science Tower, 515 Delaware Street S.E., Minneapolis, MN 55455, USA; 2 Department of Chemistry, Hansraj College, University of Delhi, Delhi 110007, India; tarunasingh@hrc.du.ac.in; 3 IMpact Biomaterials Lab, Division of Restorative Science, Minnesota Dental Research Center for Biomaterials and Biomechanics (MDRCBB), School of Dentistry, University of Minnesota, Minneapolis, MN 55455, USA; tristennies1@gmail.com (T.N.); graceliumn@gmail.com (G.S.L.); maycoll.a.johnson@gmail.com (M.J.); mei00033@umn.edu (D.M.); gu003809@umn.edu (W.G.); isha.mutreja@gmail.com (I.M.); 4 Bioinspired Oral Biomaterials and Interfaces Lab (BOBI), Department of Odontology Materials Science and Engineering, Barcelona Research Center in Multiescale Science and Engineering (CMEM), Barcelona East School of Engineering (EEBE), Universitat Politècnica de Catalunya-BarcelonaTech (UPC), 08019 Barcelona, Spain; conrado.japaricio@uic.es; 5 Catalan Institution for Research and Advanced Studies (ICREA), 08010 Barcelona, Spain; 6 Department of Developmental and Surgical Sciences, University of Minnesota, Moos Health Science Tower, 515 Delaware Street S.E., Minneapolis, MN 55455, USA; rsjones@umn.edu

**Keywords:** metal silicate, mesoporous metal silicate, nanoparticles, design of experiments, nanorods, strontium, cerium, rhodium, ruthenium

## Abstract

Mesoporous silicate nanoparticles are known for their pore size, pore volume, high surface area, and tunable surface properties and, as a result, have found application in medicine, healthcare, advanced materials, and devices. Furthermore, tailoring the silicate domain with certain metals has advanced the tissue engineering properties by moderating the cellular function at the molecular level for therapeutic response. However, limited efforts have been made to explore systemic synthesis protocols that could change structure or shape of mesoporous metal silicate nanostructures as the change in shape has inherent potential to tailor biological response. Here, we have focused on using Design of Experiments (DoE) to optimize reaction conditions for the synthesis of mesoporous strontium silicate nanostructures (MPSrSiO_2_ NSs) (nanoparticles—NPs and nanorods—NRs) with different physicochemical properties. The conditions allowed a change in shape from NPs to NRs by tailoring the concentration of liquid ammonia (liq. NH_3_, NH_4_OH, 82.7 mM to 248.2 mM) during the pre-synthesis incubation state. The MPSrSiO_2_ NPs had a significantly higher zeta potential compared to NPs (*p* = 0.0061). Also, NPs possessed significantly higher surface area (*p* = 0.0306), pore diameter (*p* = 0.0003), and pore volume (*p* = 0.0004) than NRs. In addition, the optimized reaction conditions allowed modulation of the physicochemical properties such as surface area, surface charge, amount of metal, pore diameter, pore volume, tailored degradation profile and release profile of loaded drug (gentamicin). Finally, the protocol has the advantage of allowing incorporation of other metals such as cerium, rhodium, and ruthenium independently and/or in combination.

## 1. Introduction

Since the discovery of colloidal and porous silica/silicate nanoparticles (NPs) in 1983, researchers have developed strategies to synthesize silica nanoparticles with different physicochemical properties which include solid nanoparticles (NPs), porous nanostructures (NSs), tunable size, shape, and surface chemistries [1]. These properties and approaches have played a critical role in exploring these materials for various biomedical applications and have advanced the nanomedicine, tissue engineering and regenerative medicine field, as numerous NPs-based formulations are in preclinical and clinical trials [1,2,3,4]. The current novel strategies focusing on managing and treating various bone-associated disease conditions rely on properties such as biomacromolecule delivery, bone regeneration, modulating cell function, and bioactivity which are linked to silica nanostructures as a delivery vector or active component [5]. Such nanostructures composed of mesoporous (MP) silica (SiO_2_) nanoparticles (NPs), when doped with metals like calcium, magnesium, and strontium, are gaining attention because of their inherent physicochemical properties and tendency to modulate biological responses [6,7,8,9,10,11,12,13,14,15]. The common physicochemical properties such as tunable size, biocompatibility, bioactivity, high surface-to-volume ratio, and high mechanical strength are of great significance for any biomedical or biological application [9]. The mesoporous silicate nanoparticles (MPSiO_2_ NPs) offer additional advance over metal, metal oxide, and/or polymeric nanoparticles. These NPs can be synthesized to have a high surface area, a large pore diameter, and uniform pore size. In addition, because of the presence of hydroxyl groups on the NPs’ surface, these can be functionalized with specific ligands; and due to their large surface area, these NSs can be loaded with model drugs as nanoadditives within the pores.

By combining and collectively harvesting these physical and chemical properties of MPSiO_2_ NPs, the innate bone regeneration has been recovered from clinically traumatic, infectious, and cancerous conditions [16,17]. In addition, silicate-based nanostructures can improve cell–cell communication, cell adhesion, growth, and proliferation for bone cell/tissue regeneration both in vitro and in vivo [18,19,20,21]. However, the shape (particle or rod) of nanomaterials can play a critical role in tailoring the biological response locally as they can optimize the particle’s physicochemical properties, such as total surface area, loading capacity, release profile, and degradation profile [22]. On the one side, there have been reports that focused on metal-based mesoporous silicate composite scaffolds, e.g., cobalt–chromium, steel, and titanium, to enhance the load-bearing capability. In addition, the modification or loading of such scaffolds with active drug tends to augment osteogenic differentiation and induction of mesenchymal stem cells (MSCs) with localized delivery [16]. The potential of MPSiO_2_ NPs for remineralization has led to investigation into their application in bone-related diseases or defects [23]. Based on the size and/or surface-active functional group of the nanoparticulate system, the NPs have the potential to target and deliver the payload at different sites within the human body, including craniofacial structures [1,24,25,26]. In addition, there have been efforts to design injectable hydrogel and bioink constructs for harvesting osteogenic capabilities either by releasing loaded molecules like microRNA or doped metals with MPSiO_2_ NPs [27,28]. Furthermore, gold nanoparticles loaded with mSiO_2_ NPs not only stimulated the anti-inflammatory response but also accelerated bone formation in a coculture defect in an *in vivo* model [29].

On the other side, similarly, MPSiO_2_ nanorods (NRs) with cone-shaped pores and yolk–shell nanostructures (NSs) composed of NiCuFe_2_O_4_@mSiO_2_ have found applications in protein delivery and nano-catalysis [22,30]. The doping of MPSiO_2_ NSs with metal types such as calcium (Ca), magnesium (Mg), silver (Ag), and strontium (Sr) has shown additional benefits in osteogenic and regenerative applications [9,22,28,31,32,33,34,35,36,37]. The synthesis conditions that guide metal-based MPSiO_2_ nanostructures towards nanoparticles (NPs, spherical) vs. nanorods (NRs) morphologies, and their effective porosity remained unexplored.

The objective of our study was to perform a systematic exploration of the effects of the different synthesis parameters on the structural features of these particles. For that, we used a Design of Experiments (DoE) approach and investigated the potential reaction conditions or factors that influence the transition from particles to rods in a colloidal suspension. The different shapes of NSs are potentially known to provide microenvironment signals to cells and may alter the degradation profile or rates as well as may affect the strontium and loaded nanoadditive release profile. We also examined the impact of shape on the physicochemical properties of these Sr-doped MPSiO_2_ nanostructures, including size, surface charge (zeta potential), surface area, pore diameter, and degradation profile. Furthermore, we examined how both NPs and NSs affect the loading and release of a potential nanoadditive, gentamicin, which could be added to MPSiO_2_ to mitigate infection and improve osteoinduction.

## 2. Materials and Methods

### 2.1. Materials

The required chemical reagents such as tetraethyl orthosilicate (TEOS, Si(OC_2_H_5_)_4_, product code: 86578-1L), hexadecyltrimethylammonium bromide (CTAB, CH_3_(CH_2_)_15_N(Br)(CH_3_)_3_, product code: H6269-1kg), strontium nitrate (Sr(NO_3_)_2_, product code: 243426-500g), cerium nitrate hexahydrate (Ce(NO_3_)_3_·6H_2_O, product code: 238538-500g), ruthenium chloride hydrate (RuCl_3_·xH_2_O, product code: 206229-5g), and rhodium chloride hydrate (RhCl_3_·3H_2_O, product code: 206261-5g) were purchased from Sigma Aldrich USA (St. Louis, MO, USA). Liquid ammonia 28–30% (liq. NH_3_, NH_4_OH, product code: BDH3014-500ml) ACS grade was purchased from VWR chemicals USA (Radnor, PA, USA). The stock solutions for each reagent were prepared in DI water.

### 2.2. Methods

#### 2.2.1. Synthesis of Mesoporous Strontium Silicate Nanostructures (Design of Experiments, DoE Approach)

The strontium (Sr)-doped MPSiO_2_ nanostructures were synthesized using a modified protocol based on previous publications [9,38]. In brief, the known amount of CTAB was mixed with a predetermined volume of H_2_O and liq. ammonia (liq. NH_3_, NH_4_OH). The solution was stirred for 30 min, followed by the addition of TEOS (150 µL aliquots every 30 s, final volume as per the table by DoE), and powdered strontium nitrate salt was successively added over the period of 30 min. The reaction was allowed to complete at room temperature for the next four hours. Afterward, the nanostructures (NSs) were centrifuged at 3500 rpm in 50 mL centrifuge tubes (about 35 mL at a time), and dried at 60 °C overnight. The dried NSs pellet was powdered with a mortar and pestle and then calcined at 550 °C for 5 h in an oven. The NSs thus obtained were stored at room temperature prior to any characterization and analysis. Based on the previous publication and preliminary data [9,38], we selected the reaction conditions to design experiments using a full 2^4^ factorial (1st repeat) and a fractional 2^4-1^ factorial (2nd and 3rd repeats) model. As per the model, the DoE software (version 13, by Stat-Ease Inc., Minneapolis, MN, USA) suggested 16 (full 2^4^) and 8 (fractional 2^4-1^) experimental conditions to investigate the effect of conditions on response factors such as surface area, pore diameter, pore volume, nanostructure size and shape. The conditions investigated have been included in Table S1 (Supplementary Information, SI) for repeat 1, a full 2^4^ factorial model—16 reaction conditions. The final reaction mixture volume was 200.0 mL in a 500.0 mL round-bottom flask. The consecutive repeats 2 and 3 were a fractional 2^4-1^ factorial with 8 experiments (Table 1) with common reaction conditions with repeat 1. Additional sample characterization has been included in SI.

#### 2.2.2. Gentamicin Loading and Release Study

For the gentamicin loading and release study, a 20 mg/mL gentamicin solution in phosphate-buffered saline (PBS, 1×, pH 7.4) was prepared. Then, 3 mg of each sample type was weighed in 5.0 mL clear tubes and mixed with 3.0 mL of gentamicin solution for overnight incubation (37 °C, 24 h loading). After loading, the samples were centrifuged at 5000 rpm for 10 min and supernatant was carefully removed. The MPSrSiO_2_ NSs loaded with gentamicin were dried at 37 °C and used within 24 h for the release study in PBS (1×). At each collection time (up to day 5), the tubes were centrifuged, and the supernatant (3.0 mL) was carefully collected for UV-visible spectroscopy analysis and refreshed with PBS (1×, 3.0 mL)—left at 37 °C with gentle shaking. A gentamicin (absorbance collected at lambda max: 256 nm as per literature) standard curve (included in SI as Figure S1) was used for the calculation of drug released at each time point [39]. The respective nanostructure solution incubated for the same time duration was used as a blank for UV absorbance measurement. 

#### 2.2.3. Transmission Electron Microscopy (TEM) for Size

For transmission electron microscopy (TEM) imaging, the NSs solution was prepared in ethanol, and a 6 µL suspension was dried on a copper grid. The images were collected using an FEI Tecnai G2 Spirit BioTWIN (by FEI company, acquired by Thermo Fisher Scientific, Waltham, MA, USA) or an FEI Tecnai G2 F30 field emission gun TEM (by FEI company, acquired by Thermo Fisher Scientific, Waltham, MA, USA) at the Characterization facility (Charfac) at the University of Minnesota Twin Cities Campus. The TEM images for additional reactions with different amounts of strontium nitrate (4.75 g and 9.5 g) have been included in the Supplementary Information (SI) file as Figures S2 and S3, respectively.

#### 2.2.4. Surface Charge and Brunauer–Emmett–Teller (BET) Analysis

For surface charge analysis, the particles were dispersed in distilled water (1 mg/mL) and analyzed with a Malvern Nano ZetaSizer ZS, by Malvern Panalytical, Westborough, MA, USA (University of Minnesota Nano Center, Physics and Nanotechnology Building) using a disposable U-shaped cuvette with gold electrodes. For surface area, pore diameter, and pore volume characterization of the prepared nanostructures under different reaction conditions, nitrogen (N_2_) physisorption isotherm analysis was conducted on a Quantachrome Autosorb-i instrument by Anton Paar, Ashland, VA, USA (Chemical Engineering department, University of Minnesota Twin Cities Campus). For analysis, 25–30 mg powdered samples were degassed in quartz tubes in automated mode (heating at 300 °C for 4 h at a 5 °C per minute temperature ramp rate) under vacuum followed by running the set conditions for N_2_ isotherm using ASiQwin software v3.0 (Anton Paar, Ashland, VA, USA) for data collection and analysis [9].

#### 2.2.5. Inductively Coupled Plasma-Optical Emission Spectroscopy (ICP-OES) Analysis

The different sample types were weighed in transparent plastic tubes (5.0 mL) and redispersed in 3.0 mL of Tris-HCl at pH 7.4, also used as a blank for ICP-OES analysis. At the time of collection, the tubes were vortexed and NPs suspension was centrifuged at 5000 rpm for 10 min. The 3.0 mL supernatant was carefully pipetted out and refreshed with 3.0 mL Tris-HCl buffer. For ICP-OES analysis, 100 µL stock HNO_3_ (12 M) was added to each sample tube, which was vortexed to dissolve the solute in the buffer. The samples were transferred to 15.0 mL centrifuge tubes and the volume of the solution was spiked to 10.0 mL using 2% HNO_3_ solution in Tris-HCl buffer. The samples were analyzed using the iCap 7600 Duo Inductively Coupled Plasma-Optical Emission Spectrometer (ICP-OES) system by Thermo Fisher Scientific, Waltham, MA, USA (Research Analytical Laboratory, Crops Research building, University of Minnesota, St. Paul, MN, USA).

#### 2.2.6. Design of Experiments (DoE) and Statistical Analysis

To design the experiments, we decided to investigate 4 factors with 2 levels, namely, CTAB amount (g, 1.1 g and 2.2 g), liq. NH_3_ volume (NH_4_OH, mL, 2 mL and 6 mL), TEOS volume (mL, 5 mL and 10 mL), and Sr(NO_3_)_2_ mass (g, 4.75 g and 9.5 g). Based on this information, DoE picked a regular two-level factorial design (images included in Supplementary Information, SI). For the first set or repeat, we decided to run 16 experiments (2^4^, manageable to run) suggested by the model as well with the combination of factors, and for the 2nd and 3rd repeat, ran 8 experiments (suggested by model 2^(4-1)^) as shown in Figure S5 in SI. The data collected through different analyses (a total 3 of repeats, R1—a full factorial 2^4^ model—16 reactions, while R2 and R3, each a fractional 2^4-1^ model with 8 reactions) was fed into the table generated by DoE software and analyzed. The DoE software also ran the statistical analysis using an ANOVA model and data has been included in SI. The raw data was also statistically analyzed using GraphPad Prism v10 by GraphPad Software LLC. The data was analyzed first to confirm parametric distribution and comparison amongst the groups was performed using ANOVA, with Tukey’s multiple comparison tests. The 95% confidence intervals have been used as error bars in all the graphs/data sets. The asterisk representation is as follows: non-significant (ns) for *p* > 0.05, * for *p* ≤ 0.05 (a), ** for *p* ≤ 0.01 (b), *** for *p* ≤ 0.001 (c), and **** for *p* ≤ 0.0001 (d).

## 3. Results and Discussion

### 3.1. Synthesis and Characterization

**Figure 1:**
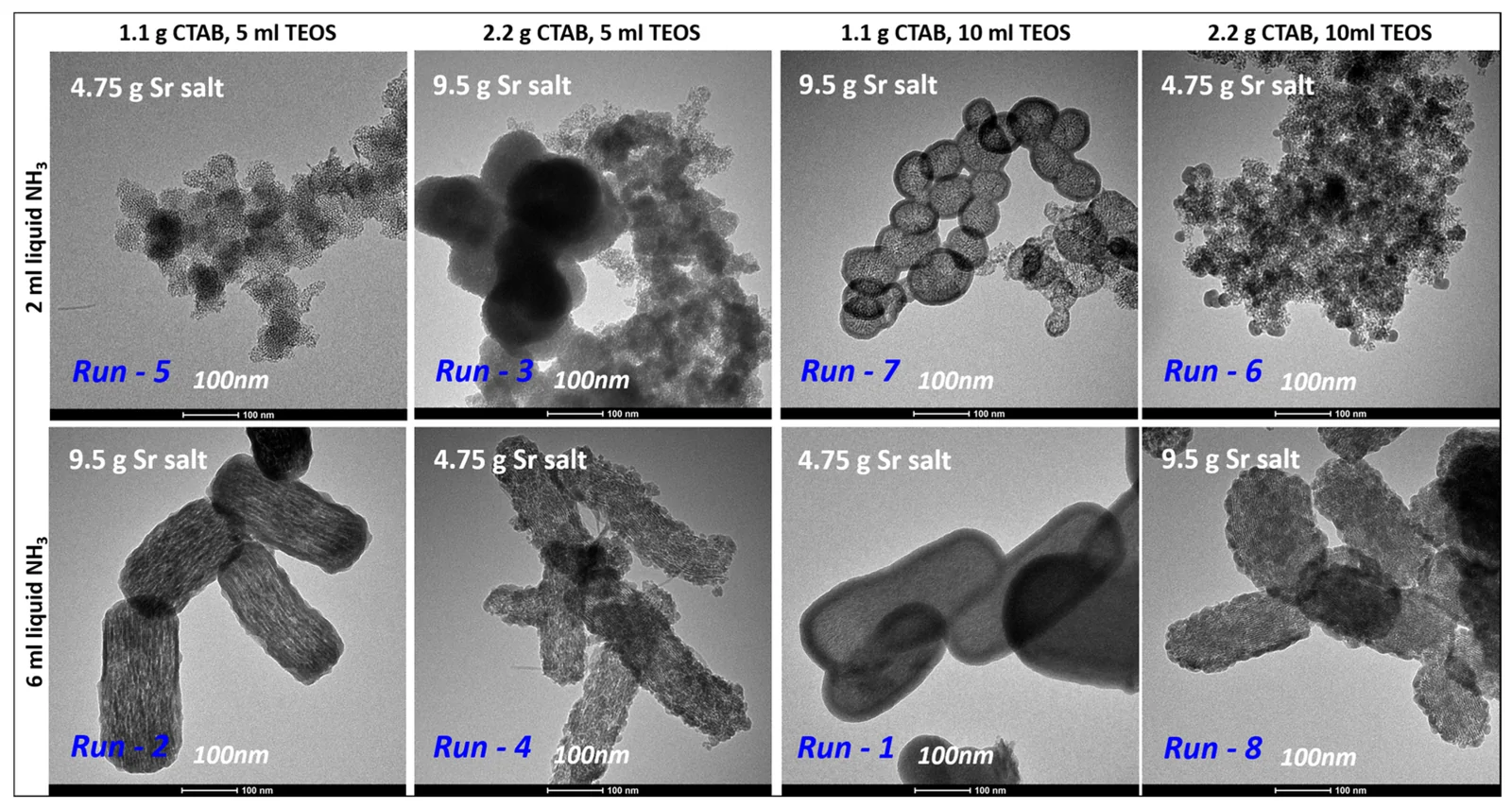
TEM image for Sr-doped MP silicate NSs (NPs-1st row and NRs-2nd row) synthesized using 8 reaction conditions with varying parameters. TEM for additional conditions (8 more) has been included in SI (total 16 conditions).

The strontium (Sr)-doped MPSiO_2_ NSs were synthesized using a modified protocol as described in the literature [9]. The CTAB (1.1 or 2.2 g) above the critical micelle concentration with liquid ammonia NH_4_OH (2.0 mL (82.7 mM) or 6.0 mL (248.2 mM)) was used in aqueous suspension to form expected templates followed by serial addition of other components such as tetraethyl orthosilicate (TEOS, 5.0 or 10.0 mL with injection of 150 µL every 30 s) and solid powdered strontium nitrate (4.75 or 9.5 g using a disposable spatula every 20 s). The nanostructures were collected by centrifugation in 50.0 mL tubes, dried at 60 °C overnight, and then calcined at 550 °C for 5 h. The synthesized and calcined mesoporous strontium silicate (MPSrSiO_2_) nanostructures (NSs) were characterized by transmission electron microscopy for size and shape, as shown in Figure 1. The TEM imaging suggested that irrespective of shape and size, all the nanostructures were mesoporous with different pore diameters, pore volumes, and surface areas (discussed in the next section). However, the shapes from this approach resulted in nanoparticles (spherical, perpendicular axes/diameters, d1 = d2) and nanorods (perpendicular axes/diameters, d1 < d2) irrespective of the amount of strontium salt used. The ratio of D2/D1 represents the aspect ratio of nanostructures. The shape of NSs was predominantly decided by the concentration of liquid ammonia (NH_4_OH) as shown in Figure 1. The self-assembly of CTAB-like surfactant molecules in aqueous medium is entropy driven, and the transition from spherical shape at the critical micelle concentration to a worm or rod shape can be driven by changes in ionic strength (addition of liq. ammonia) or incorporation of additives such as TEOS or metal salts [40]. The extended TEM characterization of full factorial (16 experiments) has been included in the Supplementary Information as Figures S2 and S3 (which have replicates from Figure 1). The TEM images for batch/repeat 2 have been added as Figure S9. The TEM imaging analysis showed that the shape of mesoporous strontium silicate was dependent on the concentration of liquid ammonia. In our previous study, calcium-, calcium–strontium-, and strontium-doped mesoporous silicate nanoparticles were characterized using Fourier transformation infrared spectroscopy (FTIR) in attenuated total reflectance (ATR) mode [9]. The analysis suggested the presence of absorption bands at 796 cm^−1^ and 1060 cm^−1^ for Si-O-Si bond stretching and bending movements, respectively. In addition, a slight blue shift was observed with metal doping within the mesoporous silicate nanostructures [9].

### 3.2. Surface Charge and Brunauer–Emmett–Teller (BET) Nitrogen Isotherm

**Figure 2:**
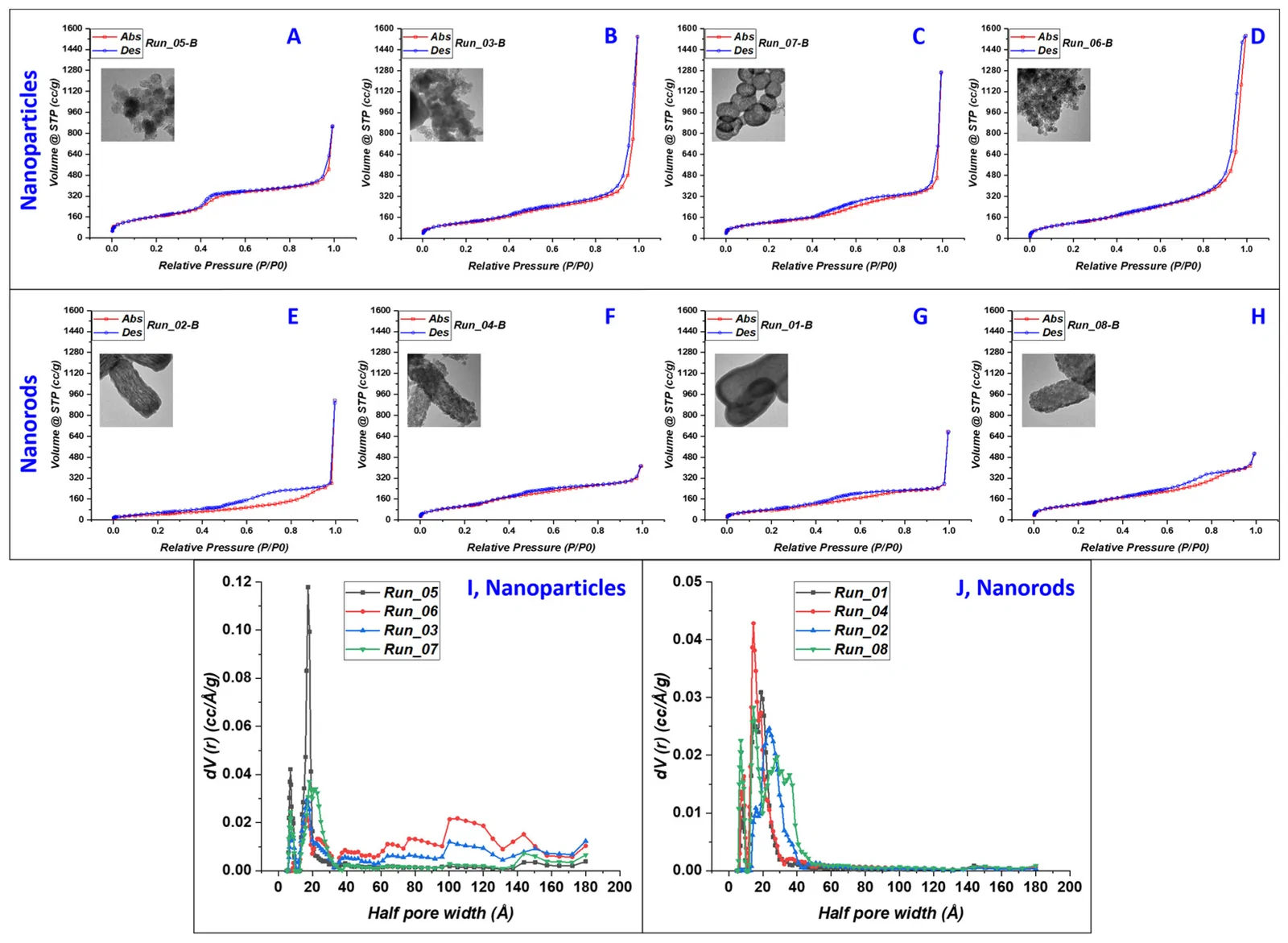
Absorption and desorption graphs for each reaction (**A**–**H**) along with pore diameters (**I**,**J**) of prepared nanostructures, compared between nanoparticles and nanorods. The inset images in A to H show the structure for the respective NS for the isotherm graph.

**Figure 3:**
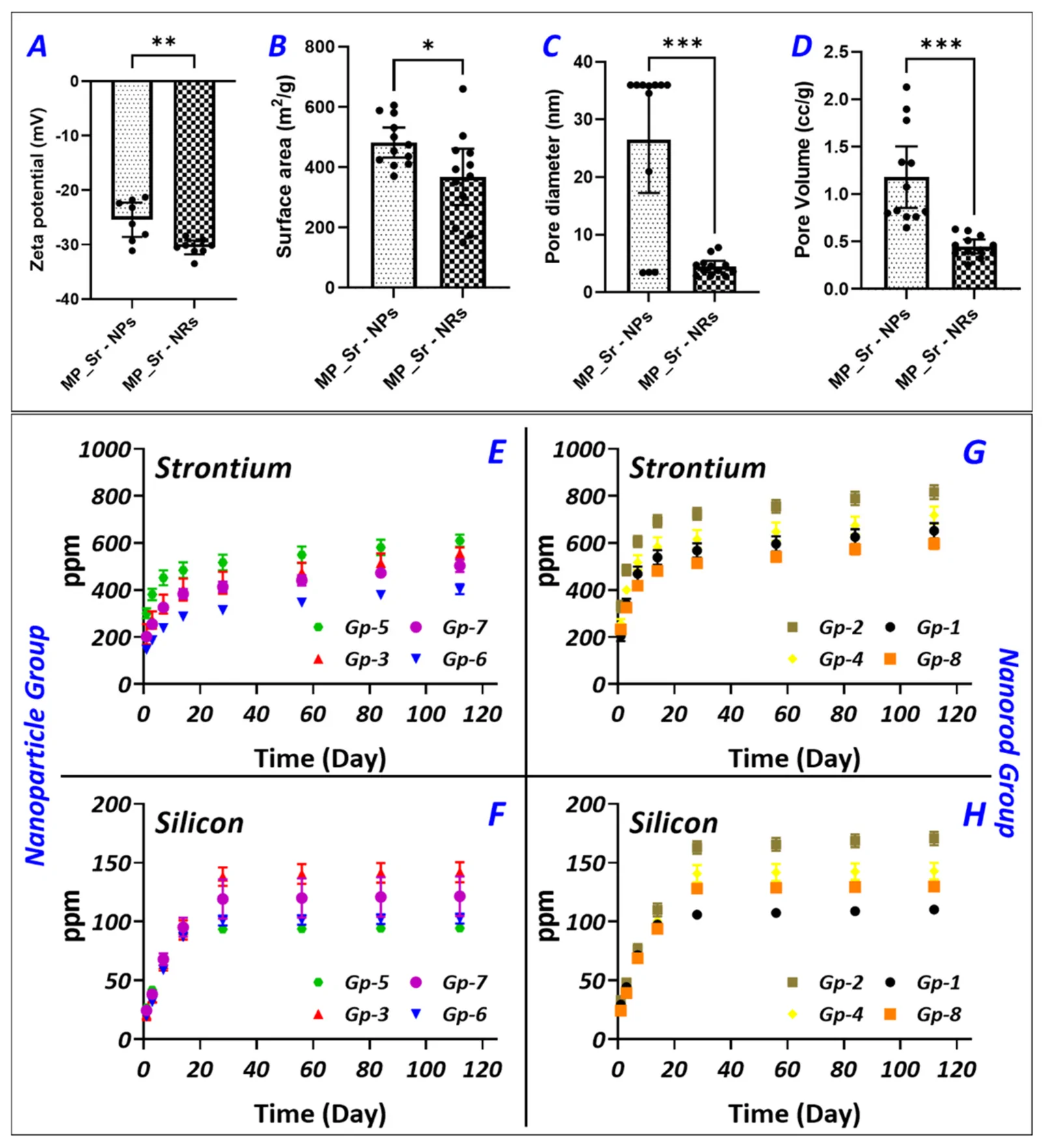
Physicochemical properties such as zeta potential (**A**), surface area (**B**), pore diameter (**C**), and pore volume (**D**) compared for NPs and NRs as nanostructures. Also, release of strontium metal (**E**,**G**) and silicon (**F**,**H**) release from each nanostructure type on degradation when exposed to Tris-HCl buffer at 7.4 up to day 112. (non-significant (ns) for *p* > 0.05, * for *p* ≤ 0.05, ** for *p* ≤ 0.01, *** for *p* ≤ 0.001).

Zeta potential analysis of Sr-doped MPSiO_2_ NSs (mesoporous (MP) strontium silicate nanostructures, MPSrSiO_2_) suggested that the surface charge on the nanostructures remained between −21.85 mV and −31.80 mV, as shown in Table 2. However, when we compared the surface charge of MPSrSiO_2_ NPs (average Zeta = −25.43 mV) with NRs (average Zeta = −30.50 mV), the NRs had a significantly lower surface charge (two-tailed *t*-test, *p* = 0.0061). Interestingly, this difference in surface charge was significant despite both NPs and NRs having the same average amount of strontium salt and TEOS. In general, the zeta potential or surface charge correlates to the stability of nanostructures. With increasing surface charge (towards the positive scale), the nanostructures (NSs not stabilized with polymer) tend to be unstable [41]. The nitrogen physisorption isotherm was used to characterize the surface area, pore volume, and pore size. A typical adsorption and desorption curve for nitrogen for each run has been included as Figure 2. For the calculation of surface area, the desorption and absorption graph was selected and BET—multipoint BET plot was selected [42]. The shape of curves in Figure 2 (A to H) suggests the pore diameter to be in the mesoporous pore diameter range. Typically, the structures/materials with pore diameter less than 2 nm are known to be microporous, while an average pore diameter between 2 nm and 50 nm constitutes mesoporosity; and macroporous materials are known to have pores with diameter larger than 50 nm [43,44]. The pore size distribution graphs are included in Figure 2I,J. Figure 2I,J clearly demonstrate that both NPs and NRs exhibit a reduced number of pores within the microporous range, predominantly displaying pores in the mesoporous range. In addition, the desorption-absorption graph was selected followed by selection of the DFT method to extract the pore diameter and pore volume values. The average values collected this way were further used in DoE. As per the literature, NRs should have higher surface area (factor of R-square) and pore volume (factor R-cube) than NPs because of their larger aspect ratio [42]. Contrary to that, we observed a higher surface area for NPs, which could be attributed to a smaller sample size or data points (12 in our study), the method used to calculate SA, and/or sample preparation during the analysis. Furthermore, the doping of Sr metal within the mesoporous silicate NSs might have contributed to the larger SA for NPs, which needs further investigation. Based on the data collected via BET nitrogen isotherm, NPs (run: 5, 3, 7, and 6) had, on average, significantly higher surface area, pore diameter, and pore volume compared to NRs (run: 2, 4, 1, and 8) as shown in Figure 3. Design of Experiments software data analysis has been discussed in Section 3.4, investigating the effect of reaction parameters on various response factors.

### 3.3. Degradation Profile via ICP-OES Analysis

The different nanostructures synthesized under the respective conditions were exposed to Tris-HCl buffer pH 7.4 for degradation analysis. The samples were divided into two groups, NPs and NRs, to simplify the analysis. The degradation profile, based on the amount of Sr and Si quantified by ICP-OES analysis, suggested a logarithmic fit to the data. Such a logarithmic fit analysis is known to couple the influence of multiple stress factors [45]. Based on this, we can speculate an initial accelerated degradation of nanostructures, which could be related to the surface effect either because of degradation itself or release of chemisorbed metal on the surface, followed by a more controlled or slower release of both elements (strontium (Sr) and silicon (Si)) up to 120 days. According to the analysis, NPs exhibited a slower and relatively smaller release of Sr and Si compared to NRs as shown in Figure 3. The amount of Si released from NPs varied from 23.13 ppm to 115.1 ppm for day 1 to day 112, respectively, while in the case of NRs, the amount increased to 29.11 ppm and 138.4 ppm. However, the released amount of silicon was not statistically different until day 84 (*p* = 0.0393) and day 112 (*p* = 0.0316) between NPs and NRs (data included in SI as Figure S4). Contrary to that, ICP-OES data suggested statistically significant changes in the released concentration of Sr from day 3 (*p* = 0.0402, NPs (274.2 ppm) vs. NRs (256.2 ppm)). Furthermore, the amount of Sr released from NPs increased to 518.1 ppm while for NRs, it was 695.9 ppm on day 112 (*p* = 0.0002). The Sr and Si release profiles from NPs and NRs showed the opposite trend relative to surface area, pore diameter, and pore volume, which indicates that the synthesized Sr-doped MPSiO_2_ NPs are more stable than the NRs, even though the NPs showed a slightly lower negative surface charge and a higher surface area, pore diameter, and pore volume, as conveyed in Figure 3.

### 3.4. Design of Experiments (DoE) Analysis

**Figure 4:**
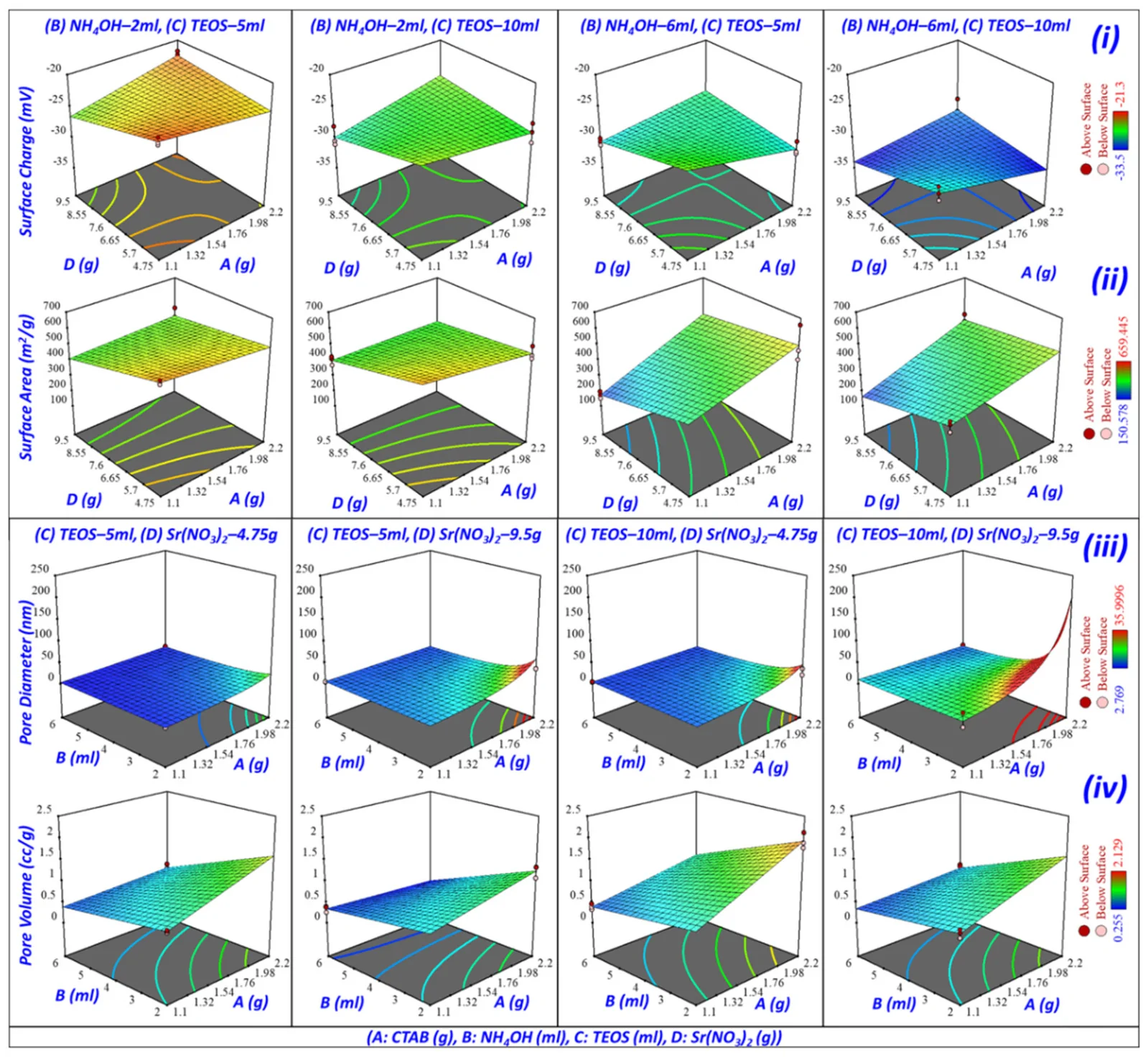
3D surface representation for zeta potential (surface charge, (**i**)), surface area (**ii**), pore diameter (**iii**), and pore volume (**iv**) data analyzed using Design of Experiments software.

**Figure 5:**
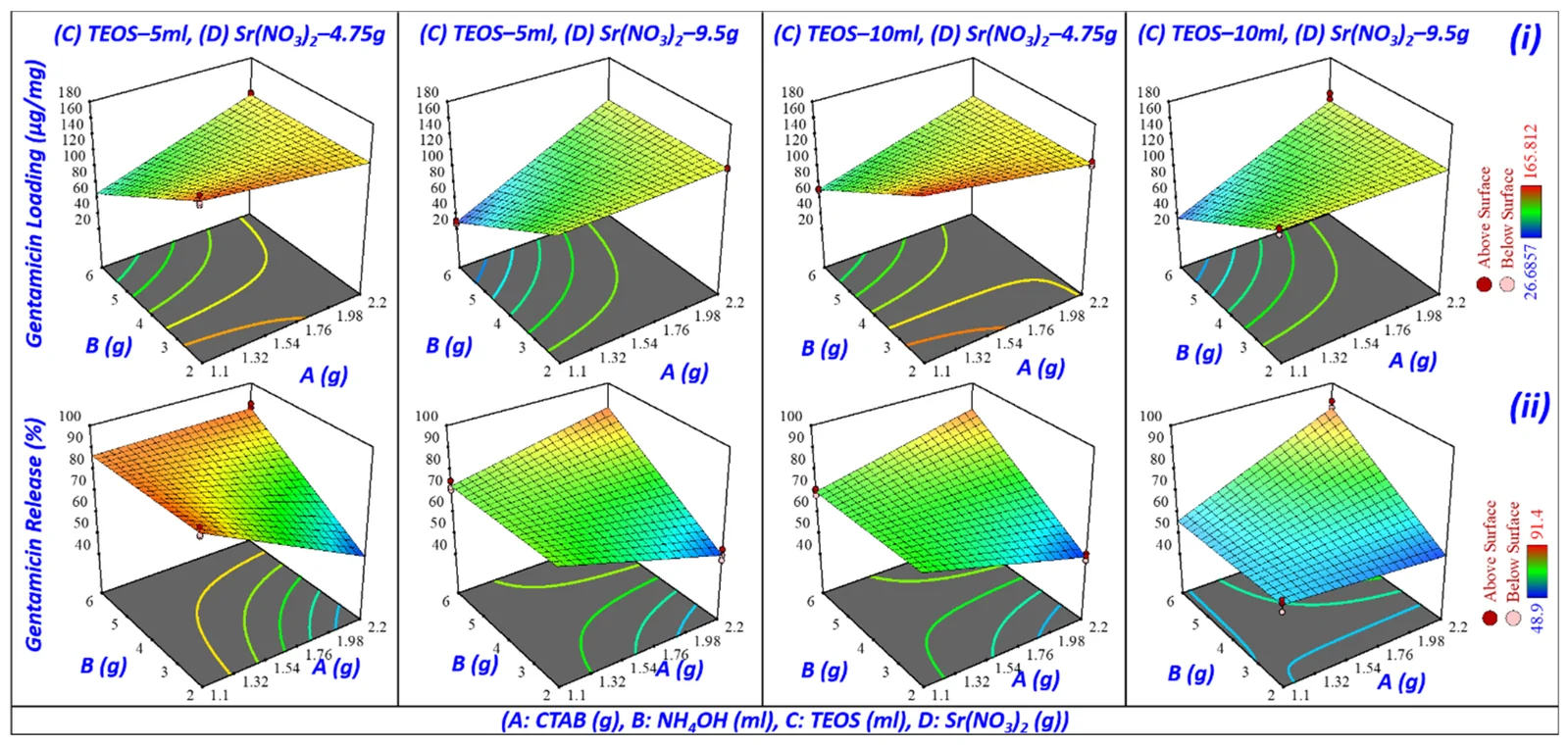
3D surface representation for loading (**i**) and release (**ii**) of gentamicin drug from nanostructures using DoE software.

The effects of CTAB (A, g) liquid ammonia (NH_4_OH, B, mL), tetraethyl orthosilicate (TEOS, C, mL), and strontium nitrate (Sr(NO_3_)_2_, D, g) on response factors such as surface charge (mV), surface area (m^2^/g), pore diameter (nm), pore volume (cc/g), gentamicin loading (µg/mg), and gentamicin release (%) were investigated using DoE software. The study was conducted using the two-level factorial design with a reduced 2FI model. The reduced 2FI model was selected to limit the study analysis dependency to first-order combinations of factors such as AB, AC, AD, BC, BD, and CD. By definition, the reduced 2FI model removes all the non-significant factors and makes the analysis simple thereby improving the statistical accuracy. For each response, delta (signal), sigma (noise), and power values were 2, 1, and 95.3%, respectively. For the simplicity of the manuscript, the data for only the factors significantly affecting the responses have been included in Figure 4 (surface charge, surface area, pore diameter, and pore volume) and Figure 5 (gentamicin loading and release). The additional conditions have been included in the SI file.

Figure 4i,ii show changes in surface charge and surface area as a function of the amount of liq. NH_3_ (B) and TEOS (C) added relative to the amount of CTAB (A) and Sr (D). The ‘Reduced 2FI’ model suggested that the AD combination had a significant effect on surface charge and surface area, with *p*-values of <0.0001 and 0.0237, respectively. The model used an ANOVA linear regression with no transformation applied to the model. The model itself was statistically significant with F-values of 30.18 and 27.31, respectively. The data suggested that as the concentration of TEOS increased, the charge reduced, which is associated with an increasing number of hydroxyl (-OH) groups, as the polycondensation reaction proceeds during either nanoparticle or nanorod formation. As shown in Figure 4i, the surface charge remained between −21.3 and −31.1 mV, with an average value of −25.43 mV (SD: 3.77) for MPSrSiO_2_ NPs, whereas for the NRs, it further reduced to −30.5 mV (SD: 1.54), which was statistically different (as shown in Figure 3A). In terms of surface area, NPs showed little variability across CTAB concentration. In contrast, NRs at lower CTAB (1.1 g) had lower surface area (~150–350 m^2^/g) irrespective of Sr amount (4.75 or 9.5 g), which increased to approximately 500 m^2^/g for CTAB 2.2 g (Figure 4ii). This increase in surface area suggests that incorporating CTAB molecules into the template would lead to more and larger mesoporous structures or canals within the nanostructures, which has been reported previously [46,47,48,49]. The model also suggested CTAB (A, *p* = 0.8012), independently and in combination with liq. NH_3_ (B, AB: *p* = 0.0673), and TEOS (C, AC: *p* = 0.9866), had some effect on the charge of nanostructures, but the effect was not statistically significant. In the case of surface area, TEOS (C) and CTAB (A or AC) did not significantly affect it, independently (*p* = 0.2333) or in conjunction (*p* = 0.4922).

Similar to surface area, pore diameter and pore volume followed a similar trend. Figure 4 shows the variability in pore diameter (Figure 4iii) and pore volume (Figure 4iv) as a function of CTAB (A) and liq. NH_3_ (B), in relation to the amount of TEOS (C) and Sr-nitrate (D). The increase in CTAB (A) concentration (1.1 g to 2.2 g) at a higher concentration of liq. NH_3_ (B), 6.0 mL, did not significantly change the pore diameter (4.48 nm, SD: 1.59), independent of increasing concentration of TEOS (C) and/or Sr-nitrate (D). The reaction condition resulted in the synthesis of mesoporous nanorods. However, when the concentration of liq. NH_3_ (B) was reduced to 2.0 mL, this led to the synthesis of mesoporous nanoparticles. There was a significant increase in pore diameter with increasing concentration of CTAB, TEOS, and Sr-nitrate, reaching 26.48 nm (SD: 14.51) as shown in Figure 4iii. The pore volume was lowest for nanorods when synthesized using 6.0 mL liq. NH_3_ but increased with an increasing amount of TEOS. This further increased significantly for nanoparticles (synthesized using 2.0 mL liq. NH_3_), with the highest being at the highest concentration of CTAB and TEOS as shown in Figure 4iv.

The surface area, pore diameter, and pore volume can sufficiently affect drug or molecular loading within the nanostructure and furthermore can tailor the release of loaded molecules. According to Christoffer G. Bavnhoj et.al., molecular loading increases with increasing surface area and decreasing pore volume because of decreasing pore diameter in mesoporous silicate, thereby influencing the drug-loading capacity dependent on the molecular size of the drug [50]. Contrary to that, in our study, gentamicin loading in both types of nanostructures, nanoparticles or nanorods, followed similar trends, as evident in Figure 5i. For Sr-doped MPSiO_2_ NPs, there was a small variability in surface area, which translated to smaller changes in gentamicin loading. Contrary to that, MPSrSiO_2_ NRs showed large variability in surface area from ~150 m^2^/g to ~650 m^2^/g, which translated to gentamicin loading of ~26 to 135 µg/mg nanorods with an average loading of ~89.51 µg/mg (SD: 44.26). In addition, the MPSrSiO_2_ NPs showed, on average, higher drug loading (136.93 µg/mg (SD: 14.15)), which was tailored based on the concentration of TEOS, Sr-nitrate and CTAB. Higher TEOS, lower Sr-nitrate and lower CTAB led to higher drug loading (143.27 µg/mg) in our study. Contrary to drug loading, MPSrSiO_2_ NRs nanorods showed faster and more drug release compared to NPs. Based on the composition, NRs released 65–90% of the drug; on the other hand, MPSrSiO_2_ NPs showed ~48 to 90% drug release, based on the composition, as can be seen in Figure 5ii. Amongst NRs, increasing the amount of CTAB from 1.1 g to 2.2 g resulted in only faster release (71.36% to 88.12%). In the case of NPs, increasing the amount of CTAB led to slower drug release (73.05% to 51.08%). The adjusted R^2^, predicted R^2^, coefficient values, F-values, and *p*-values for each response against the respective factor and model are included in tabular form as Figure S6 (for surface area and surface charge), Figure S7 (for pore diameter and pore volume) and Figure S8 (for gentamicin loading, gentamicin release, and aspect ratio) in SI. The adjusted and predicted R^2^ values (with a difference of 0.2 between adjusted and predicted) for pore volume, gentamicin loading, gentamicin release, and aspect ratio were more than 95%, suggesting the model can be used to design the conditions with selective response values. The adjusted and predicted R^2^ values (with a difference of 0.2 between adjusted and predicted) for surface area and surface charge were more than 85%, suggesting lesser dependability, and for pore diameter, the values were around 75%. 

### 3.5. Tuning the Shape

**Figure 6:**
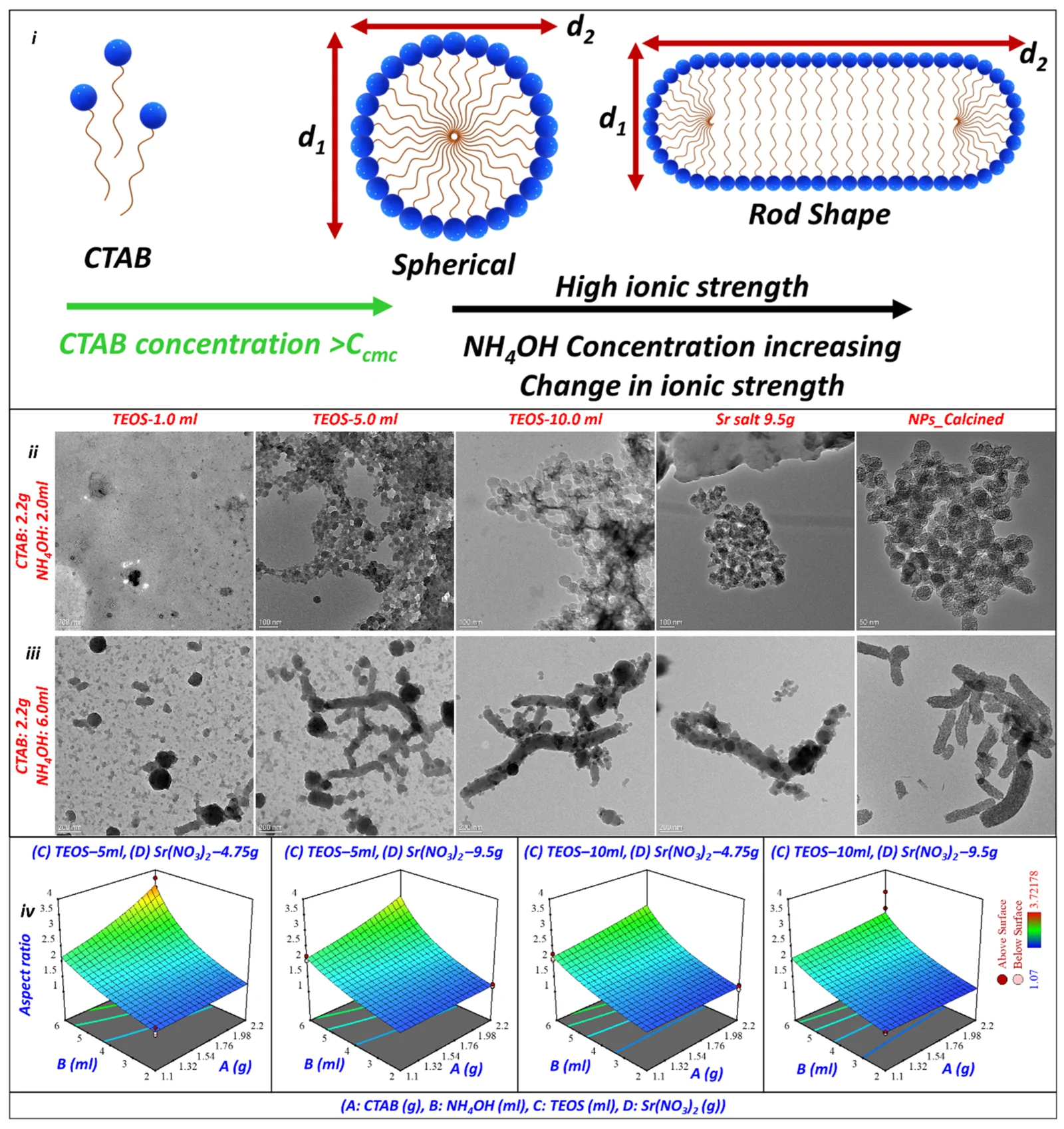
Potential growth mechanism (**i**) for nanoparticles and nanorods with a change in ionic strength of the reaction solution assisted with liq. ammonia (NH_4_OH), and figures (**ii**,**iii**) show the captured growth structure using TEM imaging. Figure (**iv**) shows the change in aspect (D2/D1) ratio represented using 3D surface graphs using DoE analysis.

The potential path to NR studied via electron microscopy has been included as Figure 6. The actual mechanism from particle to rod shape might be more critical and complicated to investigate, which needs further investigation. As per the literature, under specific conditions (hydrothermal), the nanoparticles (zero-dimensional isotropic structures, 13 nm) can self-assemble into one-dimensional anisotropic nanorods (≤100 nm) without any surfactant [51]. However, across all the experimental approaches in the DoE study, at the same reagent concentrations except for liquid ammonia (increased from 2.0 mL (82.7 mM) to 6.0 mL (248.2 mM)), we observed a change in shape from NP to NR. Figure 6i shows a schematic of the speculated formation of spherical and rod-shaped CTAB micelles as the liquid ammonia (NH_4_OH, liq. NH_3_) concentration increased. Similar reports suggesting that an increase in ammonia concentration can lead to a change in shape (particle to rod) and size (25–150 nm to 2 µm) of nanostructures have been reported elsewhere [52]. In our study, we had an unsuccessful attempt to capture the CTAB micelles prior to the addition of other additives such as TEOS; however, the earliest time point (180–210 s in reaction, 1.0 mL TEOS) showed relatively small-size seed silica NPs at a lower concentration of ammonia (82.7 mM, 2.0 mL) as shown in Figure 6ii,iii. With an increasing amount of TEOS (5.0 mL to 10.0 mL), there was an increase in size of NPs and nanorods for 82.7 mM and 248.2 mM liq. NH_3_ (NH_4_OH), respectively. The addition of strontium nitrate powder (4.75 g or 9.5 g), collection of NSs by centrifugation, and calcination did not alter the nanostructure shape, captured by electron microscopy and as shown in Figure 6. Increasing the amount of liq. NH_3_ had a significant effect on the shape, as evident in the aspect ratio (AR, D2/D1) data shown in Figure 6iv. At a lower concentration of liq. NH_3_ 2.0 mL (82.7 mM), the aspect ratio remained around 1.2 (SD: 0.068), and tailoring the concentration of CTAB (1.1 g–1.22 and 2.2 g–1.18) did not significantly change the average aspect ratio (*p* = 0.3591), suggesting no effect on nanostructure shape. In the case of Sr-doped MPSrSiO_2_ NRs, the average aspect ratio was 2.56 (SD: 0.64), and it increased significantly from 2.12 to 3.01 (*p* = 0.0197) with increasing CTAB concentration. The TEOS and Sr-nitrate concentrations had minimal effect on the aspect ratio. As per the literature, the surfactant molecules in the aqueous phase attain a spherical shape above the critical micelle concentration. As per a molecular simulation model, the hydrocarbon carbon chain of CTAB forms the micelle core because of higher hydrocarbon density [53,54]. In addition, the molecular structure of surfactant ions predominately plays a critical role in the sphere-to-rod transition, as longer carbon chain molecules provide bigger tail volume and increased hydrophobic interaction, favoring rod-like micelles [55,56]. Other shapes such as nanorods or wormlike structures have been linked to surfactant concentration, salt species, salt characteristics, and reaction conditions such as pH and ionic strength [37,40,57]. The NaBr concentration above 0.06 M resulted in rod formation along with spherical CTAB micelles [57]. Furthermore, the alkyl group chain length (C_n_, *n* = 1 to 4) in the silane is known to control the rate of hydrolysis thereby guiding the size (20 to 80 nm) of mesoporous silicate nanoparticles [58].

### 3.6. Future Directions

**Figure 7:**
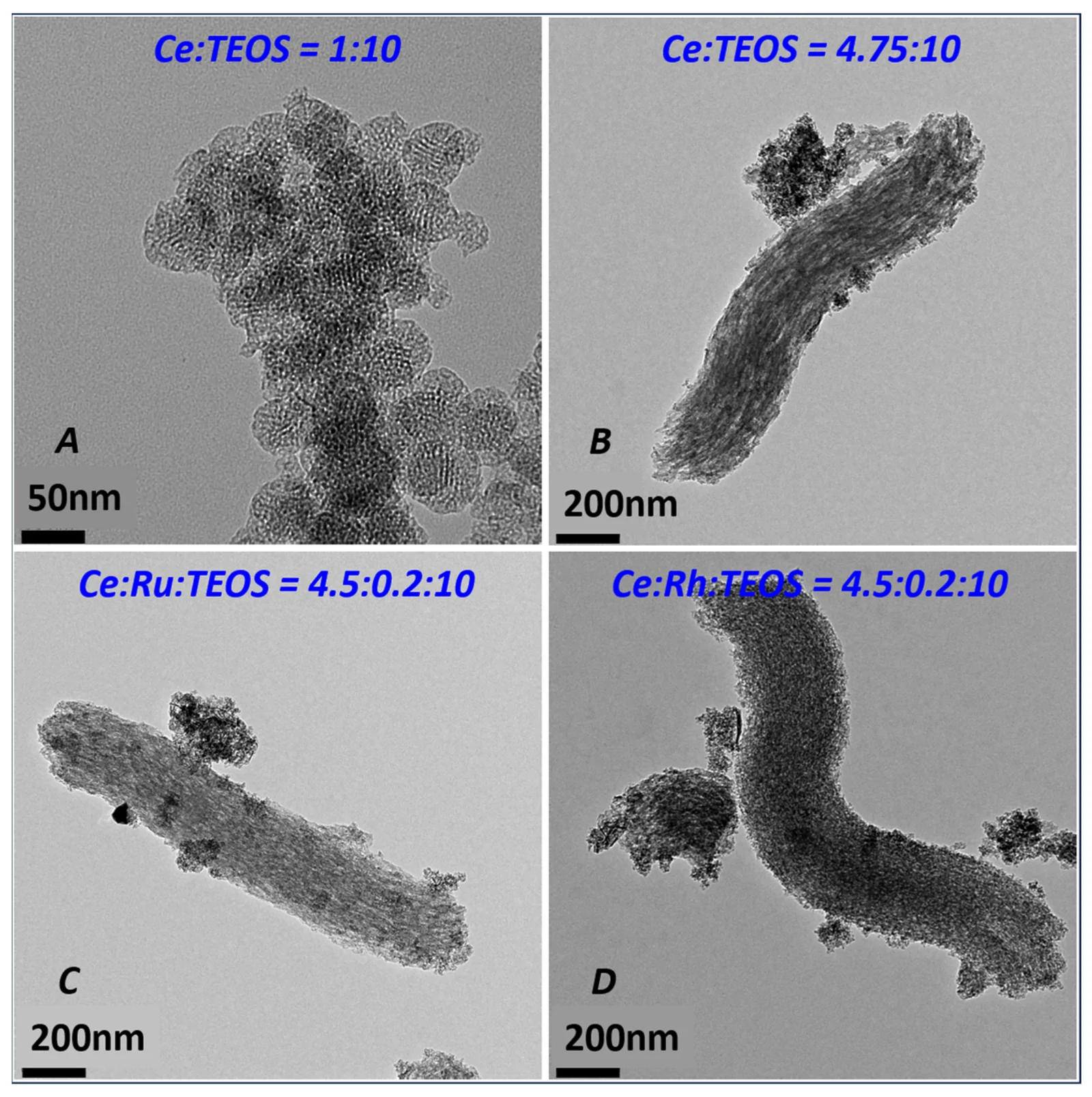
Synthesis of Ce ((**A**)-nanoparticles, (**B**)-nanorods)) and mixed cerium–ruthenium ((**C**)-nanorods) and cerium–rhodium (**D**)-nanorods) nanostructures using 2.2 g CTAB and alternate concentrations of liq. ammonia as supported in the reaction conditions.

The method motivated us to synthesize nanoparticles and nanorods with other metals and metal combinations for potential application in other fields, such as ion delivery, tissue regeneration, and catalysis. Limited literature focuses on tailoring the shape of nanostructures. According to the literature, the physicochemical properties, including NS shape, are known to modulate materials’ biological response at the molecular level [59,60,61,62]. After optimizing the conditions for MPSrSiO_2_ NPs, we synthesized mesoporous cerium silicate (MPCeSiO_2_) nanoparticles [39] and nanorods doped with other metals such as ruthenium and rhodium with the limitation of metal salt solubility (in water) in relation to the amount of TEOS or silicon (metal:silicon molar ratio less than 1). Figure 7 shows mesoporous cerium silicate nanoparticles (MPCeSiO_2_ NPs) and MPCeSiO_2_ nanorods, along with ruthenium (Ru)- or rhodium (Rh)-doped mesoporous NSs synthesized using 2.2 g CTAB and altered NH_4_OH concentration. The solubility of the metal salt played a crucial role, as with increasing concentration of metal (with respect to Si), there was an increase in aggregate formation for both types of nanostructures (particles and/or rods). Similar observations have been reported in the literature [63]. However, this method offers an edge in controlling the metal:Si molar ratios within the nanostructures, thereby tailoring the surface area, pore diameter, pore volume, loading/release profile of a model drug/molecule/ion, catalytic, and other physicochemical properties. Further, characterization of such nanostructures requires the techniques such as SEM-EDS, XPS, XRD, and FTIR for chemical characterization and elemental mapping within NSs. In a study by J. Zhang, calcium (5–10%) ions were replaced with iron (Fe) within the mesoporous calcium silicate NSs, with no cytotoxicity observed against the osteoblast cell line and improved bone regeneration and localized magnetic hyperthermia application [40]. The literature has reported doped porous metal silicate NPs with metals such as copper, magnesium, silver, and zinc and the delivery of payloads such as antibiotics, therapeutic drugs, and growth factors, both *in vitro* and *in vivo* [9,64]. When modified with polymers, lipids, and biomolecules, these MP metal silicate NSs show improved stability, biological responses, and responsiveness [65]. Highlighting the catalytic properties of porous silicate, nickel–zirconia-based mesoporous NSs supported the conversion of methane (CH_4_) and carbon dioxide (CO_2_) into syngas [66]. The application of porous materials is not limited to delivery and catalysis, but, as the porous structure allows for regulatable porosity, electron and ion transport, and high surface area, harnessing energy storage is a potential application, as suggested in review articles [43,67].

## 4. Conclusions

Strontium (Sr)-doped mesoporous silicate nanoparticles (MP-SiO_2_-NPs) were synthesized in this study using a Design of Experiments approach, and the study’s conclusions were:

- Liquid ammonia concentrations strongly influenced the shape of Sr-doped MP-SiO_2_. Increasing liquid ammonia concentrations shift MP-SiO_2_ nanostructures toward NRs.
- Sr-doped MP-SiO_2_-NPs have higher nanostructure stability than NRs and lower Sr release. In addition, the stability of NSs can be affected by size, shape, and entrapped drug.
- Future studies will examine the dynamic between stability and Sr release where specific applications may favor stability, such as restorative materials and implant coatings, whereas other applications may favor higher Sr release that can augment pulpal stem cell differentiation and regenerative bone growth.
- Higher TEOS, lower Sr-nitrate, and lower CTAB led to higher gentamicin loading. Future studies can examine the addition of different antimicrobial nanoadditives and their bioactivity.
- The ability to dope other metals (Ru and/or Rh, similar metals) can lead to the application of mesoporous metal silicate NSs in catalysis as well.

## Figures and Tables

**Table 1:** The factor conditions investigated using the Design of Experiments (DoE) software for runs (1 to 8).

Std	Run	Factor A: CTAB (g)	Factor B: Liq. NH_3_ (mL)	Factor C: TEOS (mL)	Factor D: Sr(NO_3_)_2_ (g)
4	1	1.1 (15.1 mM)	6 (248.2 mM)	10 (224.2 mM)	4.75 (106.3 mM)
2	2	1.1 (15.1 mM)	6 (248.2 mM)	5 (112.1 mM)	9.5 (224.4 mM)
3	3	2.2 (30.2 mM)	2 (82.7 mM)	5 (112.1 mM)	9.5 (224.4 mM)
6	4	2.2 (30.2 mM)	6 (248.2 mM)	5 (112.1 mM)	4.75 (106.3 mM)
1	5	1.1 (15.1 mM)	2 (82.7 mM)	5 (112.1 mM)	4.75 (106.3 mM)
8	6	2.2 (30.2 mM)	2 (82.7 mM)	10 (224.2 mM)	4.75 (106.3 mM)
7	7	1.1 (15.1 mM)	2 (82.7 mM)	10 (224.2 mM)	9.5 (224.4 mM)
5	8	2.2 (30.2 mM)	6 (248.2 mM)	10 (224.2 mM)	9.5 (224.4 mM)

**Table 2:** The surface charge values and BET analysis data in terms of surface area, pore diameter, and pore volume along with standard deviation for each run (1 to 8).

Run	Surface Charge (Unit: mV) (SD)	Surface Area (Unit: m^2^/g) (SD)	Pore Volume (Unit: cc/g) (SD)	Pore Diameter (Unit: nm) (SD)
1	(−29.45) (1.48)	341.796 (33.81)	0.389 (0.07)	3.989 (0.49)
2	(−30.65) (0.78)	173.963 (23.23)	0.343 (0.08)	4.860 (0.19)
3	(−22.50) (0.99)	447.302 (48.07)	1.247 (0.15)	35.952 (0.08)
4	(−30.10) (1.41)	536.778 (109.94)	0.535 (0.09)	3.149 (0.55)
5	(−21.85) (0.78)	591.125 (12.74)	0.792 (0.03)	3.454 (0.03)
6	(−27.75) (2.19)	486.424 (40.22)	1.934 (0.18)	30.989 (8.67)
7	(−29.60) (2.12)	401.572 (28.01)	0.745 (0.09)	35.513 (0.84)
8	(−31.80) (2.40)	418.939 (32.84)	0.530 (0.11)	5.936 (0.66)

## Data Availability

The raw data generated during the study and used for analysis and to plot the graphs can be downloaded from the DRUM repository (published online on 6th August 2026) by the University of Minnesota and can be accessed using the link: “https://doi.org/10.13020/5F90-E843” or “https://hdl.handle.net/11299/281832”.

## Supplementary Materials

The following supporting information can be downloaded at: https://www.mdpi.com/article/10.3390/jfb17100490/s1. Table S1: The table showing all the reaction conditions generated in the case of full 2^4^ factorial model by DoE. Figure S1: Gentamicin standard curve. The absorbance values collected at 256 nm at different dilutions. The inset image show absorbance values at lower dilution. The error bars are standard deviations. Figure S2: Batch 1: TEM image for samples prepared using strontium:silicon molar ratio as 0.475:1 in the reaction mixture. Figure S3: Batch 1: TEM image for samples prepared using strontium:silicon molar ratio as 1:1 in the reaction mixture. Figure S4: Amount of Sr and Si (cumulative release) released when the samples were exposed to buffer up to 112 days. Data represents an ion release comparison between NPs and NRs. Figure S5: The DoE regular two-level factorial design with respect to number of factors-top row and with potential number of experiments-first column. Table generated by DoE. Figure S6: The F, P, adjusted R^2^, predicted R^2^, and coefficient values for surface area and surface charge by DoE. Figure S7: The F, P, adjusted R^2^, predicted R^2^, and coefficient values for pore diameter and pore volume by DoE. Figure S8: The F, P, adjusted R^2^, predicted R^2^, and coefficient values for gentamicin loading, gentamicin release, and aspect ratio by DoE. Figure S9: Batch 2: TEM image for samples prepared at different reaction conditions.

## Abbreviations

The following abbreviations are used in this manuscript.

BET	Brunauer–Emmett–Teller
CTAB	Hexadecyltrimethylammonium Bromide
DoE	Design of Experiments
ICP-OES	Inductively Coupled Plasma–Optical Emission Spectroscopy
MP	Mesoporous
TEM	Transmission Electron Microscopy
TEOS	Tetraethyl Orthosilicate
NSs	Nanostructures
NPs	Nanoparticles
NRs	Nanorods
Sr	Strontium
SiO_2_	Silica/Silicate
Ru	Ruthenium
Ce	Cerium
Rh	Rhodium
NH_4_OH	Liquid Ammonia, Ammonium Hydroxide
