# Supplementary material for: Modulating the Physicochemical Properties of Mesoporous Strontium Silicate Nanostructures Using a Design-of-Experiments Approach
Source: J Funct Biomater. 2026 Sep 29;17(10):490. doi: 10.3390/jfb17100490 (PMC13705888; doi:10.3390/jfb17100490)
Supplement: Supplementary file 1 [file jfb-17-00490-s001.zip › jfb-4561533-supplementary.pdf]

Modulating the Physicochemical Properties of Mesoporous Strontium Silicate Nanostructures Using a Design-of-Experiments Approach

Dhiraj Kumar [1,*], Taruna Singh [2], Tristen Nies [3], Grace S. Liu [3], Maycoll Johnson [3], David Mei [3], Wandi Gu [3], Conrado Aparicio [4,5], Isha Mutreja [3] and Robert S. Jones [6]

[1] Division of Basic Sciences, Department of Diagnostic and Biological Sciences, University of Minnesota, Moos Health Science Tower, 515 Delaware Street S.E., Minneapolis, MN 55455, USA

[2] Department of Chemistry, Hansraj College, University of Delhi, Delhi 110007, India; tarunasingh@hrc.du.ac.in

[3] IMpact Biomaterials Lab, Division of Restorative Science, Minnesota Dental Research Center for Biomaterials and Biomechanics (MDRCBB), School of Dentistry, University of Minnesota, Minneapolis, MN 55455, USA; tristennies1@gmail.com (T.N.); graceliumn@gmail.com (G.S.L.); maycoll.a.johnson@gmail.com (M.J.); mei00033@umn.edu (D.M.); gu003809@umn.edu (W.G.); isha.mutreja@gmail.com (I.M.)

[4] Bioinspired Oral Biomaterials and Interfaces Lab (BOBI), Department of Odontology Materials Science and Engineering, Barcelona Research Center in Multiescale Science and Engineering (CMEM), Barcelona East School of Engineering (EEBE), Universitat Politècnica de Catalunya-BarcelonaTech (UPC), 08019 Barcelona, Spain; conrado.japaricio@uic.es

[5] Catalan Institution for Research and Advanced Studies (ICREA), 08010 Barcelona, Spain

[6] Department of Developmental and Surgical Sciences, University of Minnesota, Moos Health Science Tower, 515 Delaware Street S.E., Minneapolis, MN 55455, USA; rsjones@umn.edu

* Correspondence: haridhiraj@gmail.com

Table S1: The table showing all the reaction conditions generated in the case of full 2^4 factorial model by DoE.

Standard	Run/Reaction	Factor 1: CTAB (g)	Factor 2: Liq. NH_3 (ml)	Factor 3: TEOS (ml)	Factor 4: $Sr(NO_3)_2$ (g)
1	1	1.1	2	5	4.75
12	2	2.2	6	5	9.5
14	3	2.2	2	10	9.5
8	4	2.2	6	10	4.75
2	5	2.2	2	5	4.75
7	6	1.1	6	10	4.75
13	7	1.1	2	10	9.5
3	8	1.1	6	5	4.75
10	9	2.2	2	5	9.5
6	10	2.2	2	10	4.75
9	11	1.1	2	5	9.5
15	12	1.1	6	10	9.5
11	13	1.1	6	5	9.5
5	14	1.1	2	10	4.75
4	15	2.2	6	5	4.75
16	16	2.2	6	10	9.5

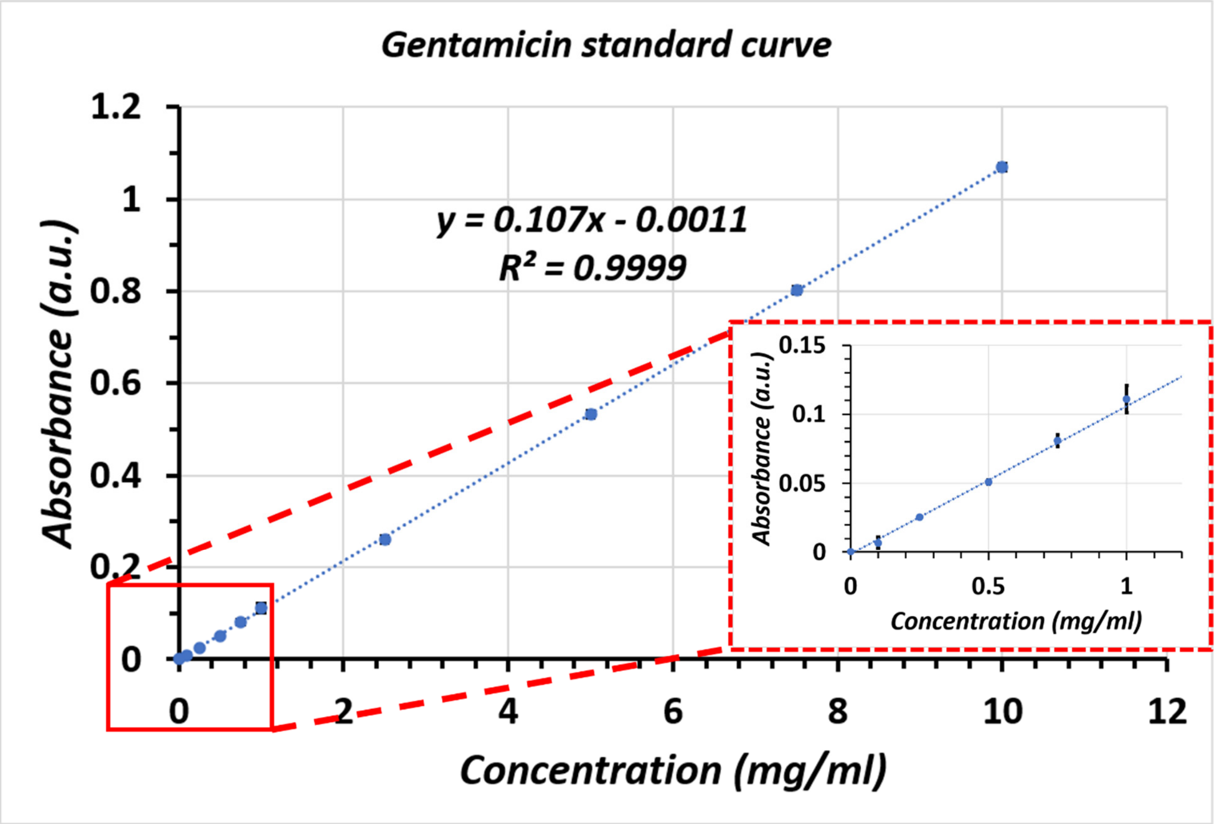

Figure S1: Gentamicin standard curve. The absorbance values collected at 256 nm at different dilutions. The inset image show absorbance values at lower dilution. The error bars are standard deviations.

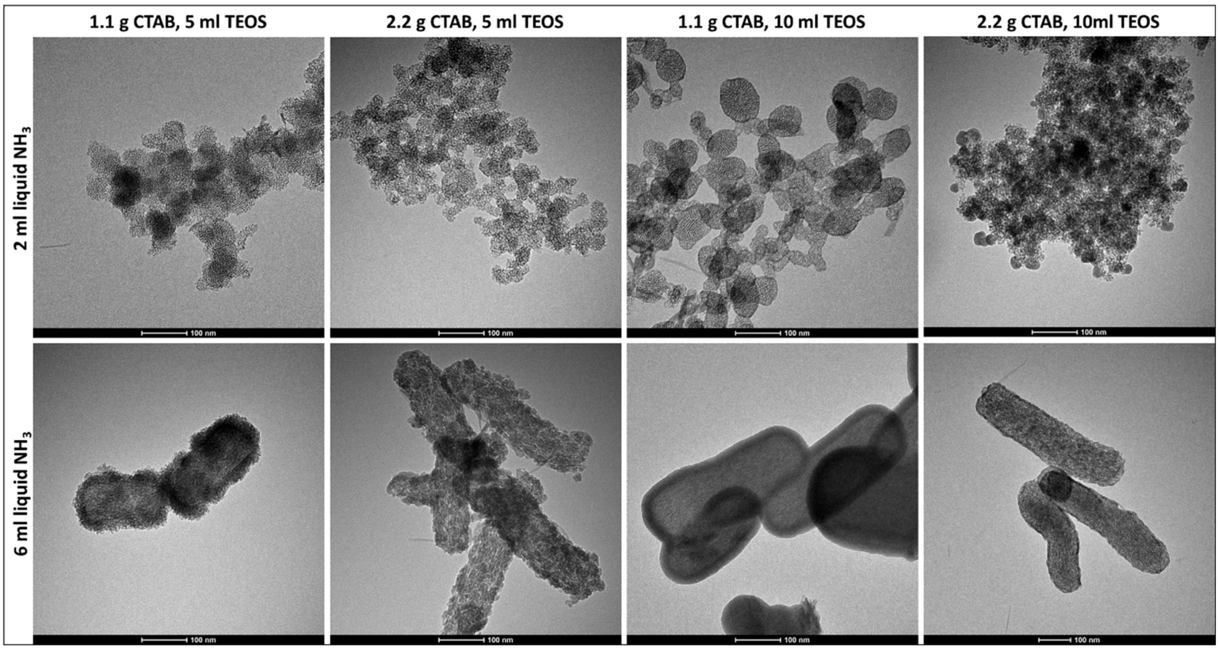

Figure S2: Batch 1: TEM image for samples prepared using strontium:silicon molar ratio as 0.475:1 in the reaction mixture.

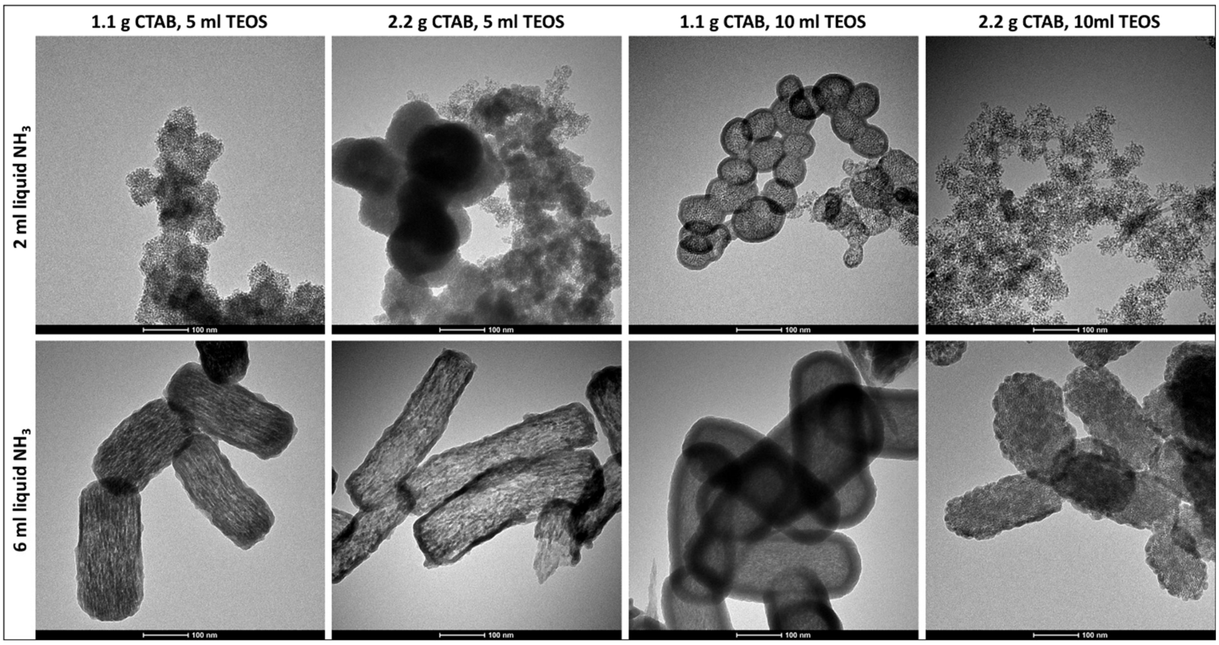

Figure S3: Batch 1: TEM image for samples prepared using strontium:silicon molar ratio as 1:1 in the reaction mixture.

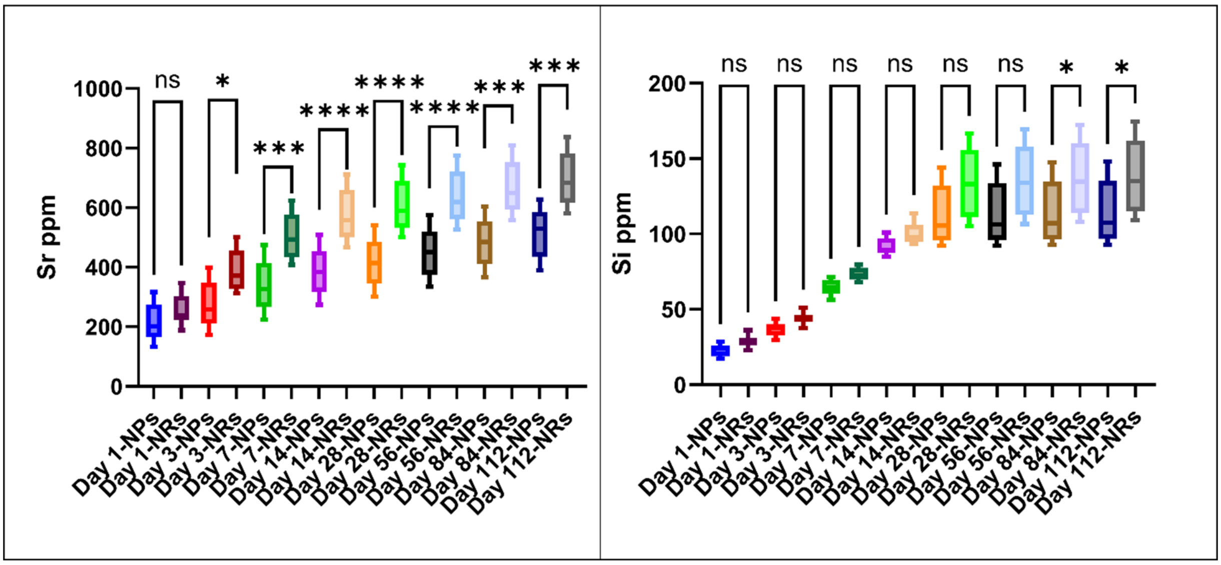

Figure S4: Amount of Sr and Si (cumulative release) released when the samples were exposed to buffer up to 112 days. Data represents an ion release comparison between NPs and NRs.

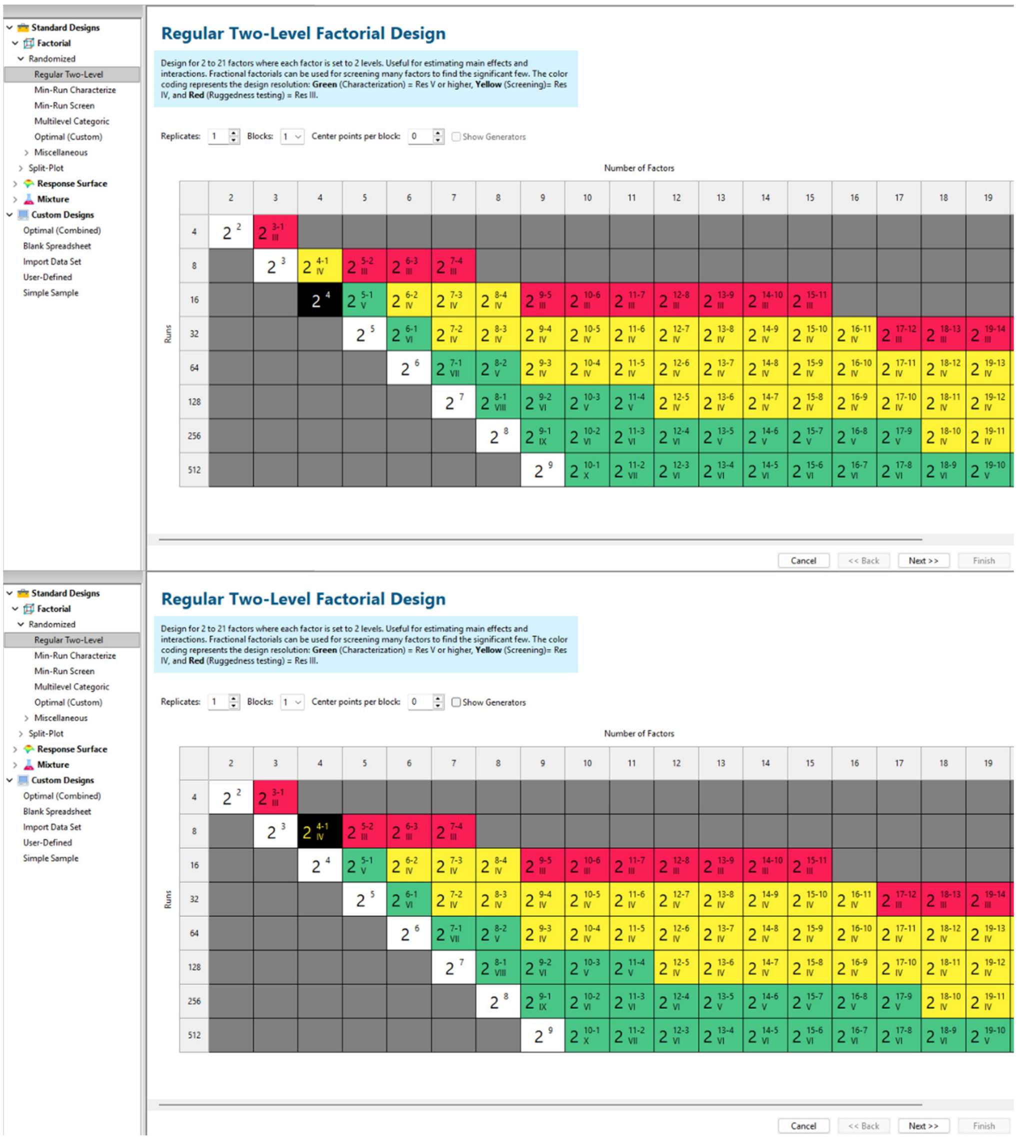

Figure S5: The DoE regular two-level factorial design with respect to number of factors-top row and with potential number of experiments-first column. Table generated by DoE.

ANOVA for selected factorial model

Response 1: Surface Area

Source	Sum of Squares	df	Mean Square	F-value	p-value	
**Model**	3.447E+05	7	49238.48	19.97	< 0.0001	significant
A-CTAB	54431.47	1	54431.47	22.08	0.0002	
B-Liq NH3	77616.29	1	77616.29	31.48	< 0.0001	
C-TEOS	3782.90	1	3782.90	1.53	0.2333	
D-SrNO3	99207.31	1	99207.31	40.24	< 0.0001	
AB	93349.30	1	93349.30	37.87	< 0.0001	
AC	1218.28	1	1218.28	0.4942	0.4922	
AD	15063.82	1	15063.82	6.11	0.0250	
**Pure Error**	39444.60	16	2465.29			
**Cor Total**	3.841E+05	23				

Factor coding is **Coded**.
Sum of squares is **Type III - Partial**

The **Model F-value** of 19.97 implies the model is significant. There is only a 0.01% chance that an F-value this large could occur due to noise.

P-values less than 0.0500 indicate model terms are significant. In this case A, B, D, AB, AD are significant model terms. Values greater than 0.1000 indicate the model terms are not significant. If there are many insignificant model terms (not counting those required to support hierarchy), model reduction may improve your model.

Fit Statistics

**Std. Dev.**	49.65		**R^2**	0.8973
**Mean**	424.74		**Adjusted R^2**	0.8524
**C.V. %**	11.69		**Predicted R^2**	0.7689
			**Adeq Precision**	14.5523

The **Predicted R^2** of 0.7689 is in reasonable agreement with the **Adjusted R^2** of 0.8524; i.e. the difference is less than 0.2.

Adeq Precision measures the signal to noise ratio. A ratio greater than 4 is desirable. Your ratio of 14.552 indicates an adequate signal. This model can be used to navigate the design space.

Coefficients | Coded Equation | Actual Equation

Coefficients in Terms of Coded Factors

Factor	Coefficient Estimate	df	Standard Error	95% CI Low	95% CI High	VIF
Intercept	424.74	1	10.14	403.25	446.22	
A-CTAB	47.62	1	10.14	26.14	69.11	1.0000
B-Liq NH3	-56.87	1	10.14	-78.35	-35.38	1.0000
C-TEOS	-12.55	1	10.14	-34.04	8.93	1.0000
D-SrNO3	-64.29	1	10.14	-85.78	-42.81	1.0000
AB	62.37	1	10.14	40.88	83.85	1.0000
AC	-7.12	1	10.14	-28.61	14.36	1.0000
AD	25.05	1	10.14	3.57	46.54	1.0000

ANOVA for selected factorial model

Response 2: Surface Charge

Source	Sum of Squares	df	Mean Square	F-value	p-value	
**Model**	302.26	7	43.18	30.18	< 0.0001	significant
A-CTAB	0.0938	1	0.0938	0.0655	0.8012	
B-Liq NH3	154.53	1	154.53	108.00	< 0.0001	
C-TEOS	69.02	1	69.02	48.24	< 0.0001	
D-SrNO3	11.34	1	11.34	7.93	0.0124	
AB	5.51	1	5.51	3.85	0.0673	
AC	0.0004	1	0.0004	0.0003	0.9866	
AD	61.76	1	61.76	43.16	< 0.0001	
**Pure Error**	22.89	16	1.43			
**Cor Total**	325.16	23				

Factor coding is **Coded**.
Sum of squares is **Type III - Partial**

The **Model F-value** of 30.18 implies the model is significant. There is only a 0.01% chance that an F-value this large could occur due to noise.

P-values less than 0.0500 indicate model terms are significant. In this case B, C, D, AD are significant model terms. Values greater than 0.1000 indicate the model terms are not significant. If there are many insignificant model terms (not counting those required to support hierarchy), model reduction may improve your model.

Fit Statistics

**Std. Dev.**	1.20		**R^2**	0.9296
**Mean**	-28.04		**Adjusted R^2**	0.8988
**C.V. %**	4.27		**Predicted R^2**	0.8416
			**Adeq Precision**	14.4317

The **Predicted R^2** of 0.8416 is in reasonable agreement with the **Adjusted R^2** of 0.8988; i.e. the difference is less than 0.2.

Adeq Precision measures the signal to noise ratio. A ratio greater than 4 is desirable. Your ratio of 14.432 indicates an adequate signal. This model can be used to navigate the design space.

Coefficients | Coded Equation | Actual Equation

Coefficients in Terms of Coded Factors

Factor	Coefficient Estimate	df	Standard Error	95% CI Low	95% CI High	VIF
Intercept	-28.04	1	0.2442	-28.56	-27.52	
A-CTAB	-0.0625	1	0.2442	-0.5801	0.4551	1.0000
B-Liq NH3	-2.54	1	0.2442	-3.06	-2.02	1.0000
C-TEOS	-1.70	1	0.2442	-2.21	-1.18	1.0000
D-SrNO3	-0.6875	1	0.2442	-1.21	-0.1699	1.0000
AB	-0.4792	1	0.2442	-0.9968	0.0384	1.0000
AC	-0.0042	1	0.2442	-0.5218	0.5134	1.0000
AD	1.60	1	0.2442	1.09	2.12	1.0000

Figure S6: The F, P, adjusted R^2, predicted R^2, and coefficient values for surface area and surface charge by DoE.

ANOVA for selected factorial model

Response 2: Pore Diameter
Transform: Inverse Sqrt
Constant: 0

Source	Sum of Squares	df	Mean Square	F-value	p-value	
**Model**	0.5361	6	0.0893	14.18	< 0.0001	significant
A-CTAB	0.0611	1	0.0611	9.70	0.0063	
B-Liq NH3	0.2446	1	0.2446	38.83	< 0.0001	
C-TEOS	0.0414	1	0.0414	6.56	0.0202	
D-SrNO3	0.0808	1	0.0808	12.83	0.0023	
AB	0.0965	1	0.0965	15.32	0.0011	
AD	0.0116	1	0.0116	1.85	0.1921	
**Residual**	0.1071	17	0.0063			
Lack of Fit	0.0047	1	0.0047	0.7421	0.4017	not significant
Pure Error	0.1024	16	0.0064			
**Cor Total**	0.6432	23				

Factor coding is **Coded.**
Sum of squares is **Type III - Partial**

The **Model F-value** of 14.18 implies the model is significant. There is only a 0.01% chance that an F-value this large could occur due to noise.

P-values less than 0.0500 indicate model terms are significant. In this case A, B, C, D, AB are significant model terms. Values greater than 0.1000 indicate the model terms are not significant. If there are many insignificant model terms (not counting those required to support hierarchy), model reduction may improve your model.

Fit Statistics

**Std. Dev.**	0.0794		**R^2**	0.8335
**Mean**	0.3902		**Adjusted R^2**	0.7747
**C.V. %**	20.34		**Predicted R^2**	0.6681
			**Adeq Precision**	9.6062

The **Predicted R^2** of 0.6681 is in reasonable agreement with the **Adjusted R^2** of 0.7747; i.e. the difference is less than 0.2.

Adeq Precision measures the signal to noise ratio. A ratio greater than 4 is desirable. Your ratio of 9.606 indicates an adequate signal. This model can be used to navigate the design space.

Coefficients Coded Equation Actual Equation

Coefficients in Terms of Coded Factors

Factor	Coefficient Estimate	df	Standard Error	95% CI Low	95% CI High	VIF
Intercept	0.3902	1	0.0162	0.3561	0.4244	
A-CTAB	-0.0505	1	0.0162	-0.0846	-0.0163	1.0000
B-Liq NH3	0.1010	1	0.0162	0.0668	0.1351	1.0000
C-TEOS	-0.0415	1	0.0162	-0.0757	-0.0073	1.0000
D-SrNO3	-0.0580	1	0.0162	-0.0922	-0.0238	1.0000
AB	0.0634	1	0.0162	0.0292	0.0976	1.0000
AD	0.0220	1	0.0162	-0.0122	0.0562	1.0000

ANOVA for selected factorial model

Response 1: Pore Volume

Source	Sum of Squares	df	Mean Square	F-value	p-value	
**Model**	6.03	7	0.8616	74.91	< 0.0001	significant
A-CTAB	1.47	1	1.47	127.52	< 0.0001	
B-Liq NH3	3.20	1	3.20	278.18	< 0.0001	
C-TEOS	0.1743	1	0.1743	15.15	0.0013	
D-SrNO3	0.2303	1	0.2303	20.02	0.0004	
AB	0.6504	1	0.6504	56.55	< 0.0001	
AC	0.1729	1	0.1729	15.03	0.0013	
AD	0.1373	1	0.1373	11.93	0.0033	
**Pure Error**	0.1840	16	0.0115			
**Cor Total**	6.22	23				

Factor coding is **Coded.**
Sum of squares is **Type III - Partial**

The **Model F-value** of 74.91 implies the model is significant. There is only a 0.01% chance that an F-value this large could occur due to noise.

P-values less than 0.0500 indicate model terms are significant. In this case A, B, C, D, AB, AC, AD are significant model terms. Values greater than 0.1000 indicate the model terms are not significant. If there are many insignificant model terms (not counting those required to support hierarchy), model reduction may improve your model.

Fit Statistics

**Std. Dev.**	0.1072		**R^2**	0.9704
**Mean**	0.8137		**Adjusted R^2**	0.9574
**C.V. %**	13.18		**Predicted R^2**	0.9334
			**Adeq Precision**	25.6949

The **Predicted R^2** of 0.9334 is in reasonable agreement with the **Adjusted R^2** of 0.9574; i.e. the difference is less than 0.2.

Adeq Precision measures the signal to noise ratio. A ratio greater than 4 is desirable. Your ratio of 25.695 indicates an adequate signal. This model can be used to navigate the design space.

Coefficients Coded Equation Actual Equation

Coefficients in Terms of Coded Factors

Factor	Coefficient Estimate	df	Standard Error	95% CI Low	95% CI High	VIF
Intercept	0.8137	1	0.0219	0.7673	0.8601	
A-CTAB	0.2472	1	0.0219	0.2008	0.2936	1.0000
B-Liq NH3	-0.3651	1	0.0219	-0.4115	-0.3187	1.0000
C-TEOS	0.0852	1	0.0219	0.0388	0.1316	1.0000
D-SrNO3	-0.0980	1	0.0219	-0.1444	-0.0516	1.0000
AB	-0.1646	1	0.0219	-0.2110	-0.1182	1.0000
AC	0.0849	1	0.0219	0.0385	0.1313	1.0000
AD	-0.0756	1	0.0219	-0.1220	-0.0292	1.0000

Figure S7: The F, P, adjusted R^2, predicted R^2, and coefficient values for pore diameter and pore volume by DoE.

ANOVA for selected factorial model

Response 1: Gentamicin loading

Source	Sum of Squares	df	Mean Square	F-value	p-value	
**Model**	36832.81	7	5261.83	199.91	< 0.0001	significant
A-CTAB	6453.66	1	6453.66	245.19	< 0.0001	
B-Liq NH3	13495.51	1	13495.51	512.73	< 0.0001	
C-TEOS	20.07	1	20.07	0.7624	0.3955	
D-SrNO3	2961.14	1	2961.14	112.50	< 0.0001	
AB	12402.17	1	12402.17	471.19	< 0.0001	
AC	74.88	1	74.88	2.84	0.1111	
AD	1425.38	1	1425.38	54.15	< 0.0001	
**Pure Error**	421.14	16	26.32			
**Cor Total**	37253.94	23				

Factor coding is **Coded**.
Sum of squares is **Type III - Partial**

The **Model F-value** of 199.91 implies the model is significant. There is only a 0.01% chance that an F-value this large could occur due to noise.

P-values less than 0.0500 indicate model terms are significant. In this case A, B, D, AB, AD are significant model terms. Values greater than 0.1000 indicate the model terms are not significant. If there are many insignificant model terms (not counting those required to support hierarchy), model reduction may improve your model.

Fit Statistics

**Std. Dev.**	5.13	**R^2**	0.9887
**Mean**	113.22	**Adjusted R^2**	0.9837
**C.V. %**	4.53	**Predicted R^2**	0.9746
		**Adeq Precision**	44.0640

The **Predicted R^2** of 0.9746 is in reasonable agreement with the **Adjusted R^2** of 0.9837; i.e. the difference is less than 0.2.

Adeq Precision measures the signal to noise ratio. A ratio greater than 4 is desirable. Your ratio of 44.064 indicates an adequate signal. This model can be used to navigate the design space.

Coefficients | Coded Equation | Actual Equation

Coefficients in Terms of Coded Factors

Factor	Coefficient Estimate	df	Standard Error	95% CI Low	95% CI High	VIF
Intercept	113.22	1	1.05	111.00	115.44	
A-CTAB	16.40	1	1.05	14.18	18.62	1.0000
B-Liq NH3	-23.71	1	1.05	-25.93	-21.49	1.0000
C-TEOS	0.9144	1	1.05	-1.31	3.13	1.0000
D-SrNO3	-11.11	1	1.05	-13.33	-8.89	1.0000
AB	22.73	1	1.05	20.51	24.95	1.0000
AC	-1.77	1	1.05	-3.99	0.4537	1.0000
AD	7.71	1	1.05	5.49	9.93	1.0000

ANOVA for selected factorial model

Response 2: Gentamicin Release

Source	Sum of Squares	df	Mean Square	F-value	p-value	
**Model**	5565.81	7	795.12	141.21	< 0.0001	significant
A-CTAB	40.72	1	40.72	7.23	0.0161	
B-Liq NH3	1873.73	1	1873.73	332.76	< 0.0001	
C-TEOS	423.86	1	423.86	75.28	< 0.0001	
D-SrNO3	266.27	1	266.27	47.29	< 0.0001	
AB	2250.41	1	2250.41	399.66	< 0.0001	
AC	396.91	1	396.91	70.49	< 0.0001	
AD	313.93	1	313.93	55.75	< 0.0001	
**Pure Error**	90.09	16	5.63			
**Cor Total**	5655.91	23				

Factor coding is **Coded**.
Sum of squares is **Type III - Partial**

The **Model F-value** of 141.21 implies the model is significant. There is only a 0.01% chance that an F-value this large could occur due to noise.

P-values less than 0.0500 indicate model terms are significant. In this case A, B, C, D, AB, AC, AD are significant model terms. Values greater than 0.1000 indicate the model terms are not significant. If there are many insignificant model terms (not counting those required to support hierarchy), model reduction may improve your model.

Fit Statistics

**Std. Dev.**	2.37	**R^2**	0.9841
**Mean**	70.90	**Adjusted R^2**	0.9771
**C.V. %**	3.35	**Predicted R^2**	0.9642
		**Adeq Precision**	27.4521

The **Predicted R^2** of 0.9642 is in reasonable agreement with the **Adjusted R^2** of 0.9771; i.e. the difference is less than 0.2.

Adeq Precision measures the signal to noise ratio. A ratio greater than 4 is desirable. Your ratio of 27.452 indicates an adequate signal. This model can be used to navigate the design space.

Coefficients | Coded Equation | Actual Equation

Coefficients in Terms of Coded Factors

Factor	Coefficient Estimate	df	Standard Error	95% CI Low	95% CI High	VIF
Intercept	70.90	1	0.4844	69.87	71.93	
A-CTAB	-1.30	1	0.4844	-2.33	-0.2757	1.0000
B-Liq NH3	8.84	1	0.4844	7.81	9.86	1.0000
C-TEOS	-4.20	1	0.4844	-5.23	-3.18	1.0000
D-SrNO3	-3.33	1	0.4844	-4.36	-2.30	1.0000
AB	9.68	1	0.4844	8.66	10.71	1.0000
AC	4.07	1	0.4844	3.04	5.09	1.0000
AD	3.62	1	0.4844	2.59	4.64	1.0000

ANOVA for selected factorial model

Response 1: Aspect Ratio
Transform: Inverse

Source	Sum of Squares	df	Mean Square	F-value	p-value	
**Model**	1.15	7	0.1641	53.49	< 0.0001	significant
A-CTAB	0.0142	1	0.0142	4.62	0.0473	
B-Liq NH3	1.08	1	1.08	352.43	< 0.0001	
C-TEOS	0.0077	1	0.0077	2.51	0.1330	
D-SrNO3	0.0031	1	0.0031	1.02	0.3265	
AB	0.0333	1	0.0333	10.85	0.0046	
AC	0.0081	1	0.0081	2.63	0.1241	
AD	0.0011	1	0.0011	0.3613	0.5562	
**Pure Error**	0.0491	16	0.0031			
**Cor Total**	1.20	23				

Factor coding is **Coded**.
Sum of squares is **Type III - Partial**

The **Model F-value** of 53.49 implies the model is significant. There is only a 0.01% chance that an F-value this large could occur due to noise.

P-values less than 0.0500 indicate model terms are significant. In this case A, B, AB are significant model terms. Values greater than 0.1000 indicate the model terms are not significant. If there are many insignificant model terms (not counting those required to support hierarchy), model reduction may improve your model.

Fit Statistics

**Std. Dev.**	0.0554	**R^2**	0.9590
**Mean**	0.6232	**Adjusted R^2**	0.9411
**C.V. %**	8.89	**Predicted R^2**	0.9078
		**Adeq Precision**	17.8707

The **Predicted R^2** of 0.9078 is in reasonable agreement with the **Adjusted R^2** of 0.9411; i.e. the difference is less than 0.2.

Adeq Precision measures the signal to noise ratio. A ratio greater than 4 is desirable. Your ratio of 17.871 indicates an adequate signal. This model can be used to navigate the design space.

Coefficients | Coded Equation | Actual Equation

Coefficients in Terms of Coded Factors

Factor	Coefficient Estimate	df	Standard Error	95% CI Low	95% CI High	VIF
Intercept	0.6232	1	0.0113	0.5992	0.6471	
A-CTAB	-0.0243	1	0.0113	-0.0483	-0.0003	1.0000
B-Liq NH3	-0.2123	1	0.0113	-0.2362	-0.1883	1.0000
C-TEOS	0.0179	1	0.0113	-0.0061	0.0419	1.0000
D-SrNO3	0.0114	1	0.0113	-0.0125	0.0354	1.0000
AB	-0.0372	1	0.0113	-0.0612	-0.0133	1.0000
AC	0.0184	1	0.0113	-0.0056	0.0423	1.0000
AD	0.0068	1	0.0113	-0.0172	0.0308	1.0000

Figure S8: The F, P, adjusted R^2, predicted R^2, and coefficient values for gentamicin loading, gentamicin release, and aspect ratio by DoE.

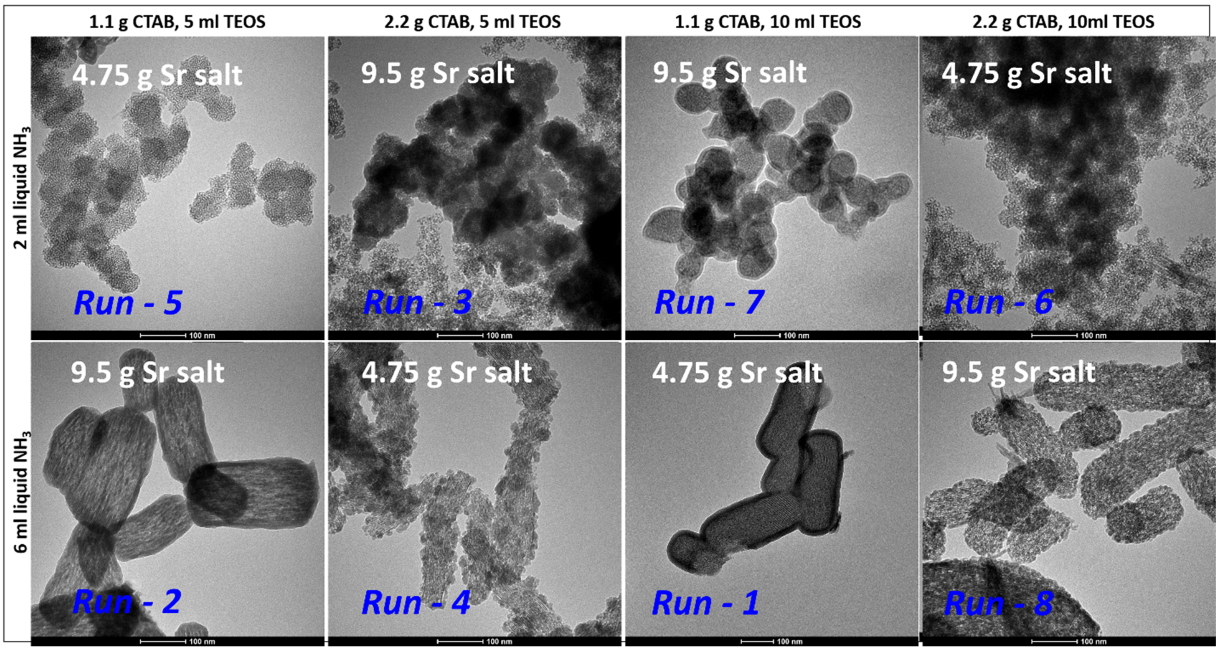

Figure S9: Batch 2: TEM image for samples prepared at different reaction conditions.
